# Supplementary material for: CRISPR activation reveals SOX5/6/9 as key transcriptional regulators directing iPSC-derived cells toward a notochordal lineage
Source: Stem Cell Reports. 2026 Aug 27;21(9):103060. doi: 10.1016/j.stemcr.2026.103060 (PMC13555567; doi:10.1016/j.stemcr.2026.103060)
Supplement: Supplementary file 1 [file mmc1.pdf]

**Stem Cell Reports, Volume *21***

## Supplemental Information

# CRISPR activation reveals *SOX5/6/9* as key transcriptional regulators directing iPSC-derived cells toward a notochordal lineage

**Xiaole Tong, Marieke Visscher, Frank M. Riemers, Danielle Versluis, Niels Geijsen, Peng Shang, Marianna A. Tryfonidou, and Deepani W. Poramba-Liyanage**

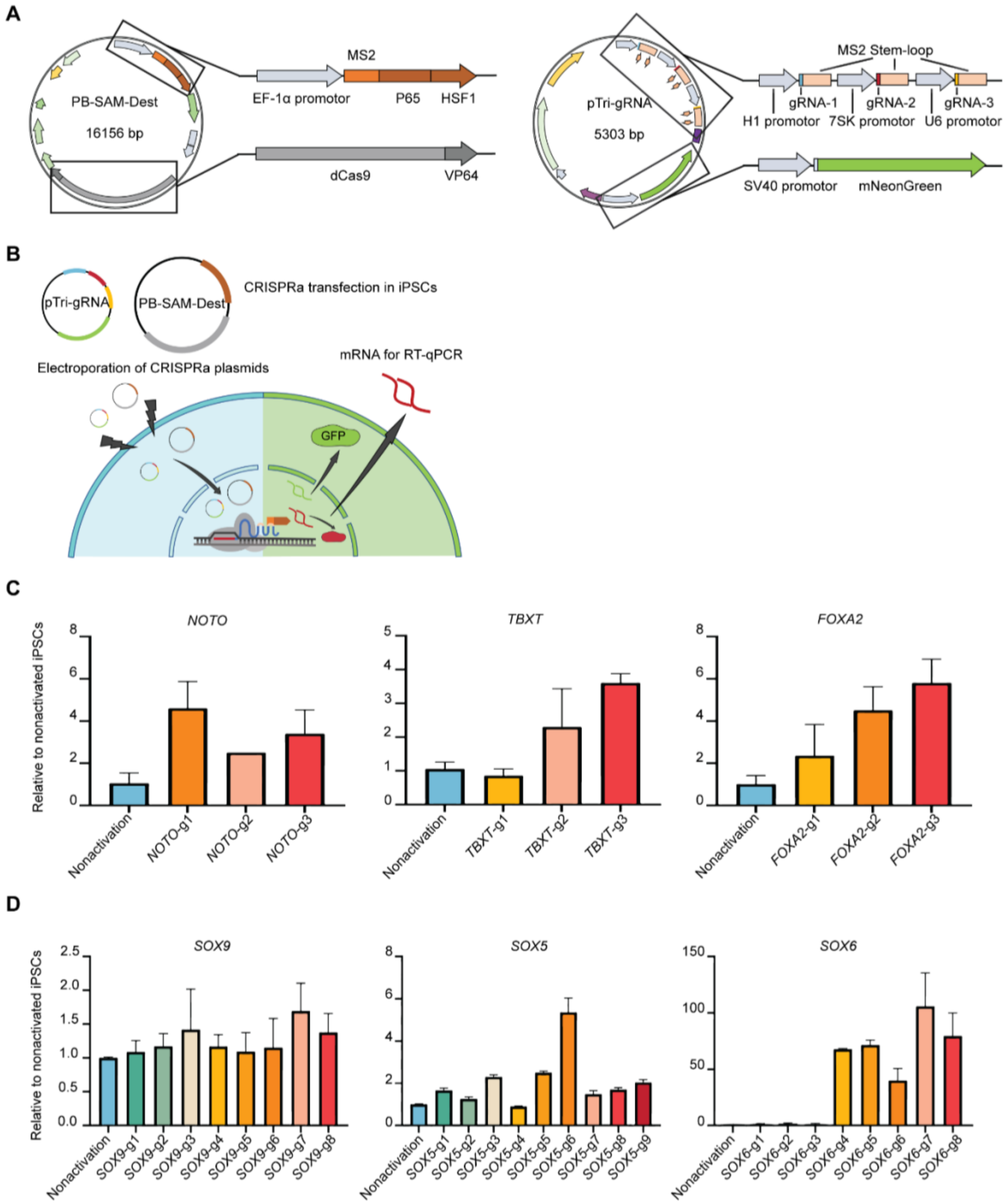


**Figure S1. CRISPRa-mediated induction of target genes in iPSCs, related to Figure 1**

(A) Schematic of constructs used to introduce the CRISPRa-SAM system and guide RNAs into cells, together with *mNeonGreen*. The CRISPR construct delivers the dCas9, and components of the CRISPRa-SAM system, the VP64 proteins, P65, HSF1 and the MS2 coat protein. The pTri-gRNA construct that allows for the expression of 3 separate guides via its promoter was used for guide delivery; with the H1 promoter expressing guide 1, the 7SK promoter expressing guide 2, and the U6 promoter expressing guide 3. An SV40 promoter that drives the expression of *mNeonGreen* is introduced with the pTri-gRNA construct.

(B) gRNA selection pipeline. Human iPSCs were introduced with the PB-SAM-Dest construct together with the pTri-gRNA construct targeting different genomic locations by electroporation, and cells were collected for RT-qPCR.

(C and D) Preliminary gRNA screening for *NOTO*, *TBXT*, *FOXA2*, *SOX9*, *SOX5* and *SOX6*. Data are presented as mean ± SD, n=2 biological replicates (independent transfections). This screen was used to rank gRNAs, whereafter selected guides were subsequently validated in triplicate with statistical analysis (see Figure 1F).

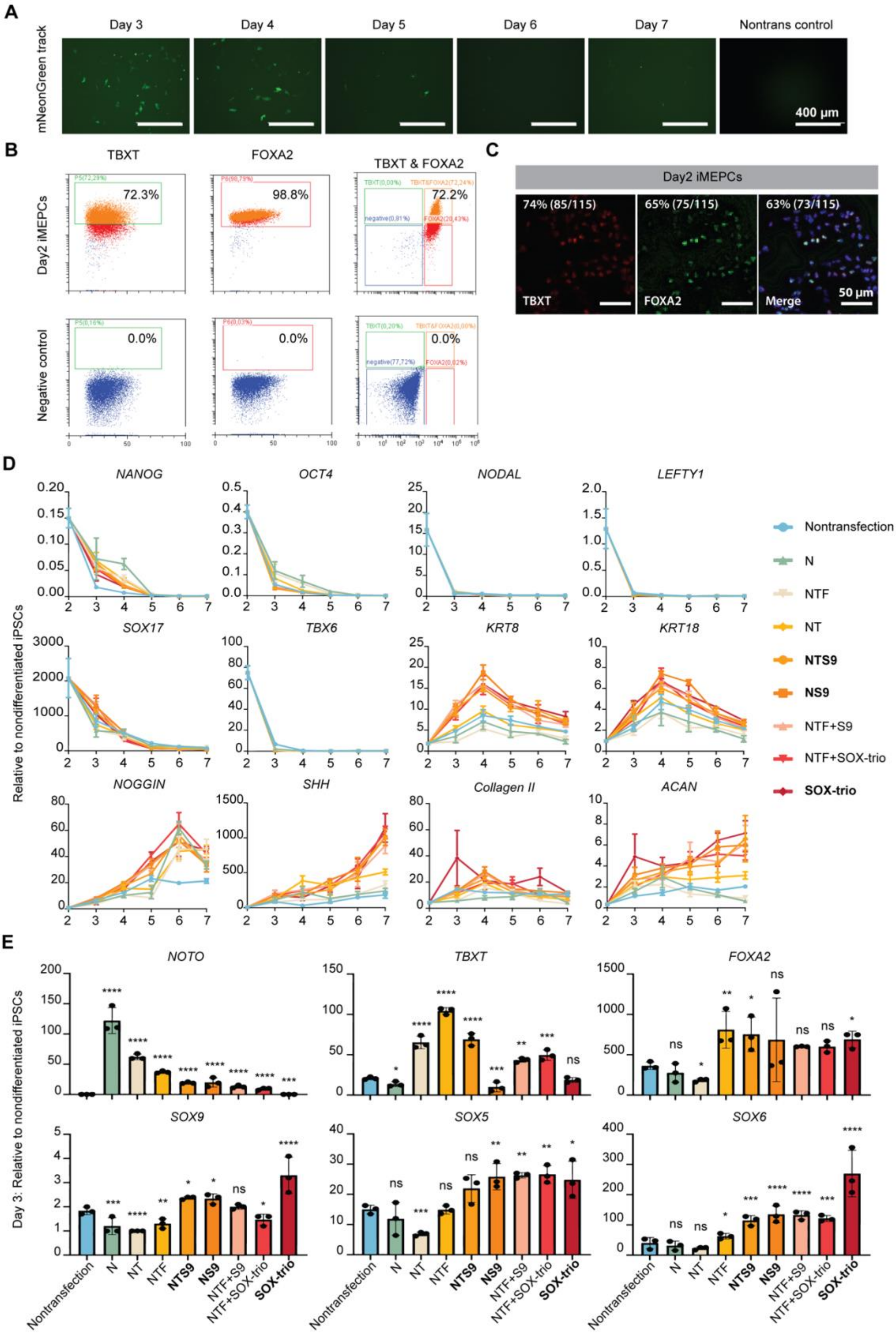

A
Day 3
Day 4
Day 5
Day 6
Day 7
Nontrans control
mNeonGreen track
400 µm
B
TBXT
FOXA2
TBXT & FOXA2
Day2 iMEPCs
Negative control
72.3%
98.8%
72.2%
0.0%
0.0%
0.0%
C
Day2 iMEPCs
74% (85/115)
65% (75/115)
63% (73/115)
TBXT
FOXA2
Merge
50 µm
D
NANOG
OCT4
NODAL
LEFTY1
SOX17
TBX6
KRT8
KRT18
NOGGIN
SHH
Collagen II
ACAN
Relative to nondifferentiated iPSCs
Nontransfection
N
NTF
NT
NTS9
NS9
NTF+S9
NTF+SOX-trio
SOX-trio
E
NOTO
TBXT
FOXA2
SOX9
SOX5
SOX6
Day 3: Relative to nondifferentiated iPSCs
Nontransfection
N
NT
NTF
NTS9
NS9
NTF+S9
NTF+SOX-trio
SOX-trio

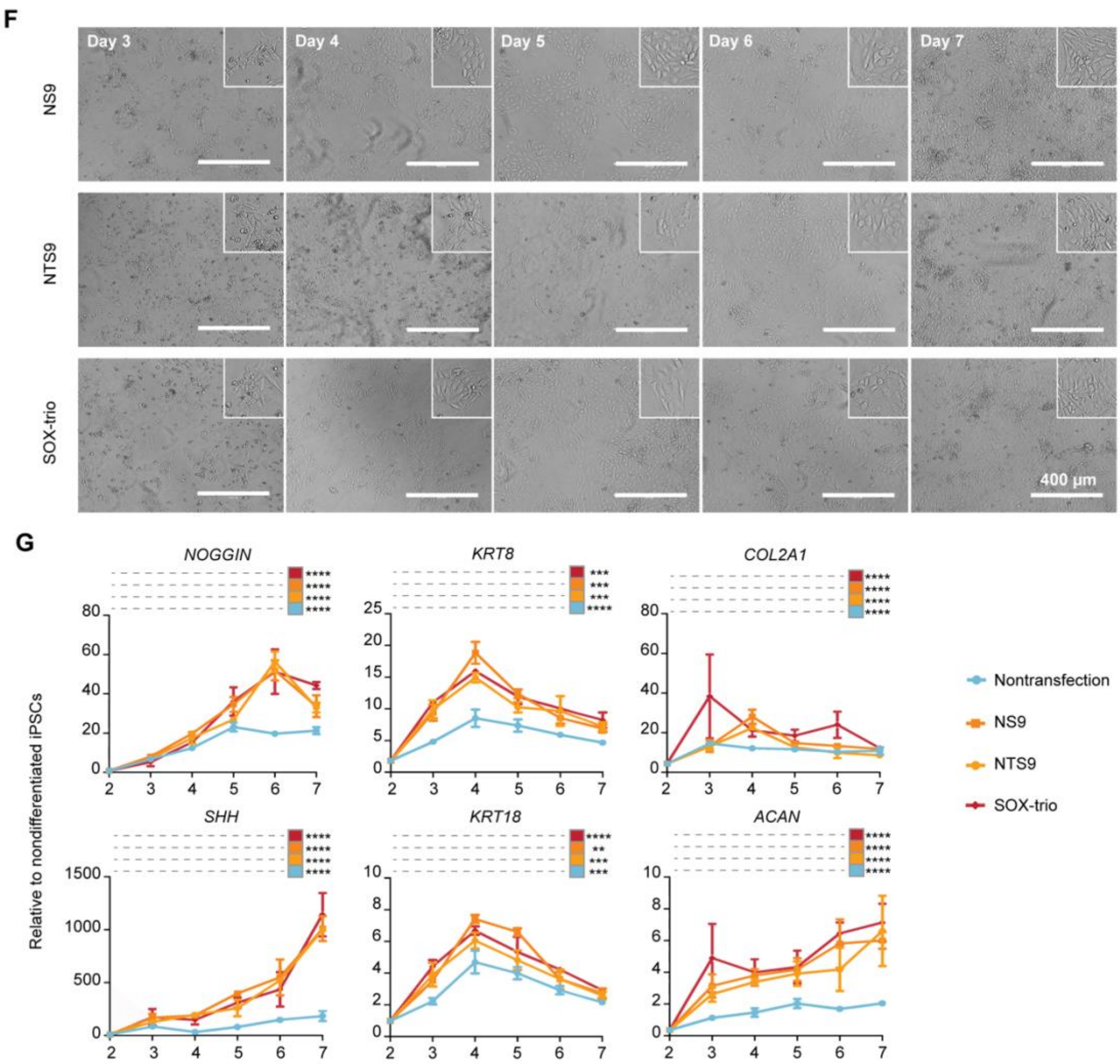


**Figure S2. CRISPRa-mediated induction of target genes to promote NC lineage commitment, related to Figure 2**

(A) mNeonGreen expression coupled to the pTri-gRNA plasmid as a readout for transfection efficiency. mNeonGreen expression remained detectable up to 5 days after transfection into iMEPCs.

(B) FACS measurement of day 2 iMEPCs showing induction of TBXT single positive; FOXA2 single positive and TBXT and FOXA2 double positive cell populations after WNT pathway activation.

(C) IF staining of day 2 iMEPCs for TBXT and FOXA2 positive populations.

(D) Time course expressions of pluripotency markers (*NANOG*, *OCT4*), WNT pathway markers (*NODAL* and *LEFTY1*), endoderm (*SOX17*), mesoderm marker (*TBX6*) and notochordal markers (*KRT8, KRT18, NOGGIN, SHH, COL2A1 and ACAN*) with comparable gene expression profiles in all groups over the culture period. Data presented as mean ± SD, n=3 biological replicates generated independently.

(E) mRNA levels of CRISPRa targeted genes in day 3 iMEPCs (24 hours after CRISPRa transfection) show successful gene activation. Significance indicated at the day 3 CRISPRa transfected cells compared with day 3 non-transfected (nontrans) cells.

(F) Cell morphology changes overtime after CRISPRa transfection in NTS9, NS9 and SOX-trio groups.

(G) Time course expressions of all key notochordal genes in Nontransfection, NTS9, NS9 and SOX-trio groups. Significance indicated at the day 7 differentiated iPSCs compared with day 2 iMEPCs. Data presented as mean ± SD, *P<0.05, **P<0.01, ***P<0.001, ***P<0.0001, ns: no significance, n=3 biological replicates generated independently.

Scalebars: 400 µm in A and F; 50 µm in C.

N: *NOTO* guides; NT: *NOTO*-g1+*TBXT*-g3; NTF: *NOTO*-g1+*TBXT*-g3+*FOXA2*-g3; NS9: *NOTO*-g1+*SOX9*-g7; NTS9: *NOTO*-g1+*TBXT*-g3+*SOX9*-g7; NTF+S9: *NOTO*-g1+*TBXT*-g3+*FOXA2*-g3+*SOX9*-g7; SOX-trio: *SOX9*-g7+*SOX5*-g6+*SOX6*-g7; NTF+SOX-trio: *NOTO*-g1+*TBXT*-g3+*FOXA2*-g3+ *SOX9*-g7+*SOX5*-g6+*SOX6*-g7

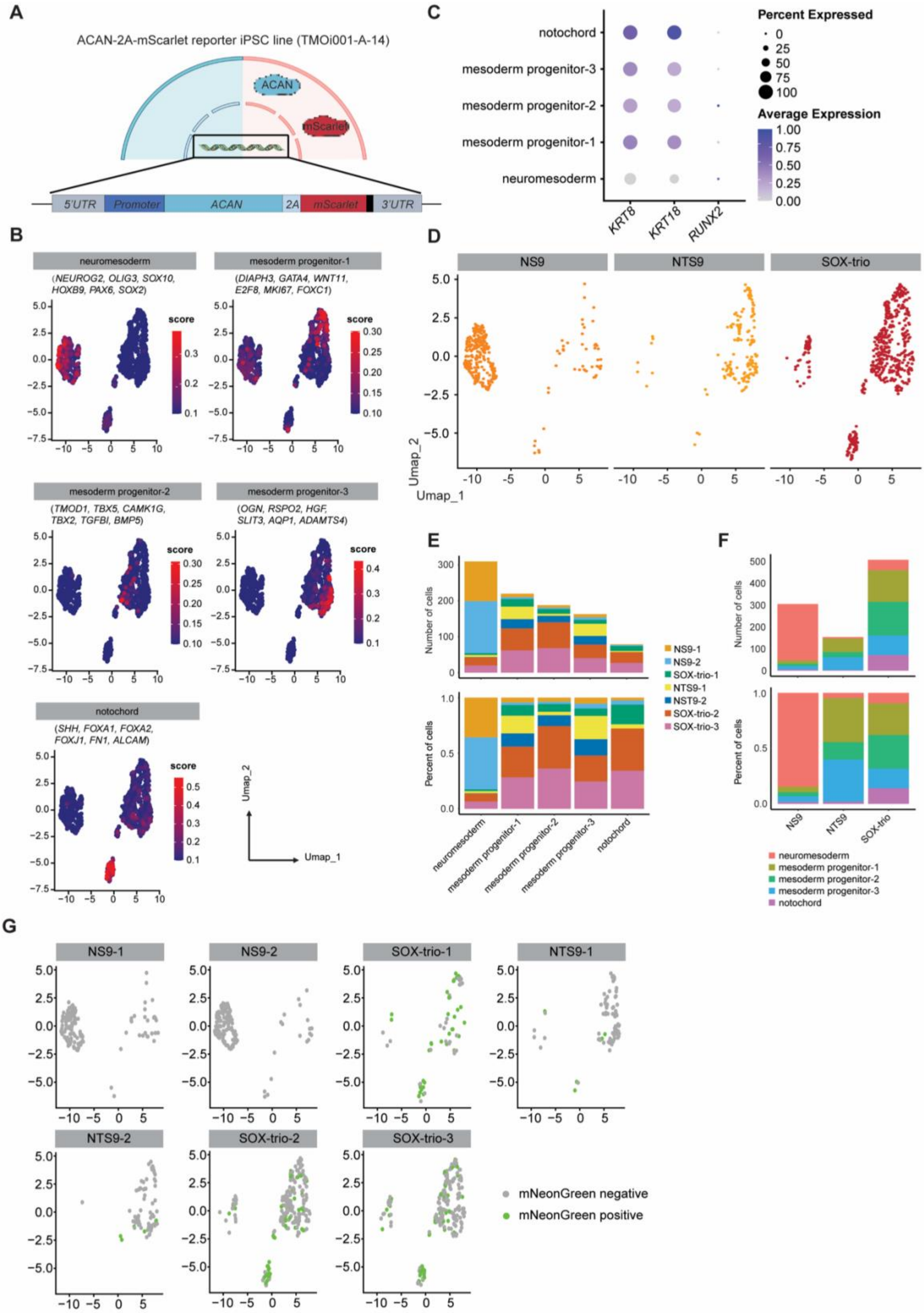
A
ACAN-2A-mScarlet reporter iPSC line (TMOi001-A-14)
ACAN
mScarlet
5'UTR
Promoter
ACAN
2A
mScarlet
3'UTR
B
neuromesoderm
(NEUROG2, OLIG3, SOX10, HOXB9, PAX6, SOX2)
mesoderm progenitor-1
(DIAPH3, GATA4, WNT11, E2F8, MKI67, FOXC1)
mesoderm progenitor-2
(TMOD1, TBX5, CAMK1G, TBX2, TGFBI, BMP5)
mesoderm progenitor-3
(OGN, RSPO2, HGF, SLIT3, AQP1, ADAMTS4)
notochord
(SHH, FOXA1, FOXA2, FOXJ1, FN1, ALCAM)
score
Umap_2
Umap_1
C
notochord
mesoderm progenitor-3
mesoderm progenitor-2
mesoderm progenitor-1
neuromesoderm
KRT8
KRT18
RUNX2
Percent Expressed
0
25
50
75
100
Average Expression
1.00
0.75
0.50
0.25
0.00
D
NS9
NTS9
SOX-trio
Umap_2
Umap_1
E
Number of cells
Percent of cells
NS9-1
NS9-2
SOX-trio-1
NTS9-1
NST9-2
SOX-trio-2
SOX-trio-3
neuromesoderm
mesoderm progenitor-1
mesoderm progenitor-2
mesoderm progenitor-3
notochord
F
Number of cells
Percent of cells
NS9
NTS9
SOX-trio
neuromesoderm
mesoderm progenitor-1
mesoderm progenitor-2
mesoderm progenitor-3
notochord
G
NS9-1
NS9-2
SOX-trio-1
NTS9-1
NTS9-2
SOX-trio-2
SOX-trio-3
mNeonGreen negative
mNeonGreen positive

**Figure S3. Single-cell RNA sequencing highlights the key contribution of the SOX-trio to NC lineage commitment, related to Figure 3**

(A) The schematic of the ACAN-2A-mScarlet reporter iPSC line (TMOi001-A-14).

(B) Identification of key clusters based on the average combined marker score using UCell.

(C) Scaled dot plots of mean normalised expression for *KRT8*, *KRT18* and *RUNX2* genes per cluster.

(D) UMAP-based plots indicating the contribution of each gRNA combination activated by the CRISPRa-SAM system with the visualized five clusters.

(E and F) Contribution of gRNA combinations and sequencing run to each cluster.

(G) UMAP plots displaying scaled *mNeonGreen* intensity (0–100) after quality filtering. For NS9 and NTS9, fewer *mNeonGreen*-positive cells passed filtering, consistent with lower retained viability. Technical replicates were generated for each TF combination. For the SOX-trio condition, two biological replicates were performed: SOX-trio-1 represents the first biological replicate, while SOX-trio-2 and SOX-trio-3 are technical replicates from the second biological replicate and were pooled for downstream analyses in Figure 3.

Technical replicates were generated for each transcription-factor combination. For the SOX-trio condition, two biological replicates were performed: the first biological replicate consisted of a single dataset (SOX-trio-1), while the second biological replicate consisted of two technical replicates (SOX-trio-2 and SOX-trio-3), which were pooled for downstream analysis.

NS9: *NOTO*-g1+*SOX9*-g7; NTS9: *NOTO*-g1+*TBXT*-g3+*SOX9*-g7; SOX-trio: *SOX9*-g7+*SOX5*-g6+*SOX6*-g7

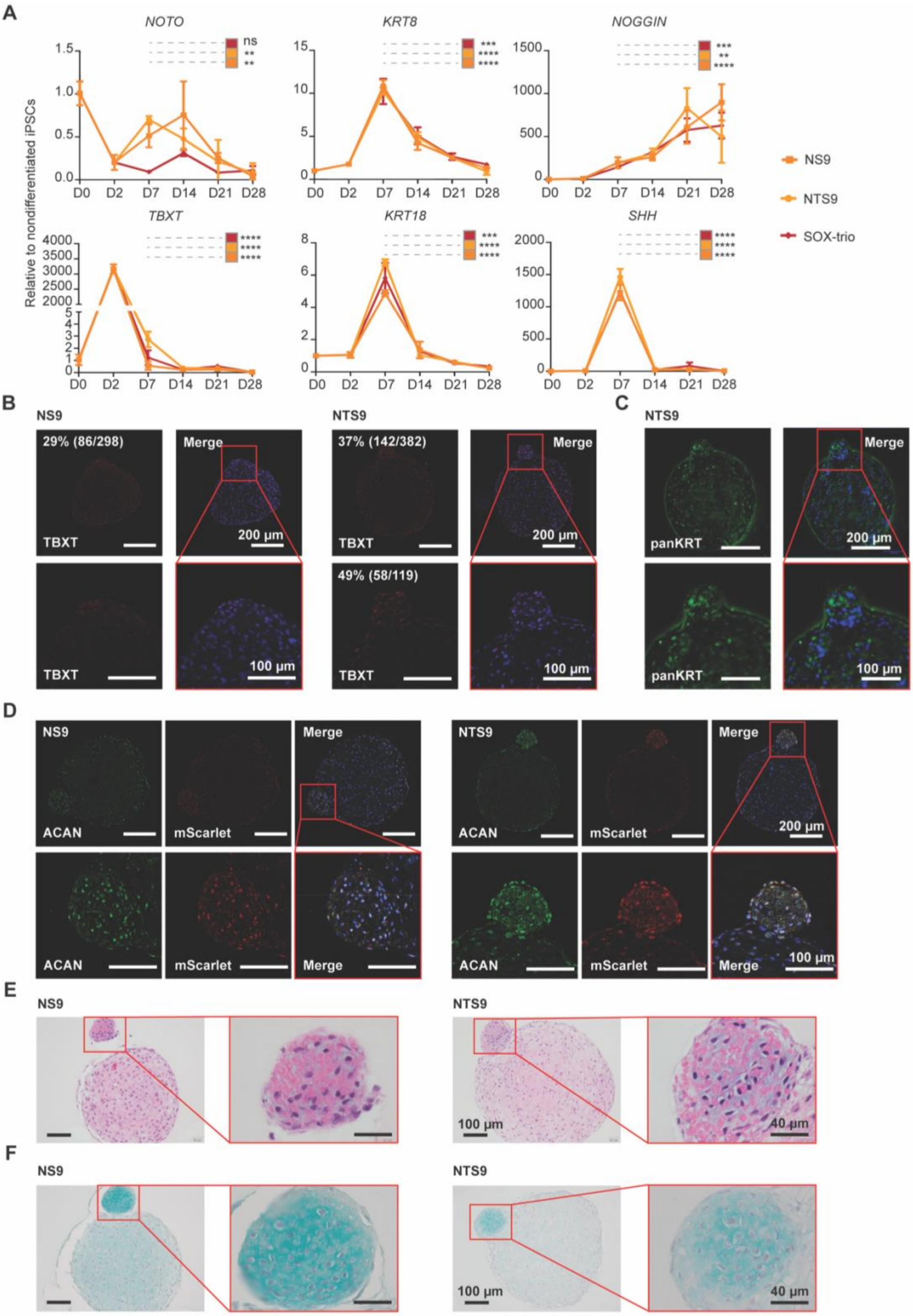


**Figure S4. Matrix deposition by iPSCs promoted to the NC lineage through CRISPRa during 3D pellet culture, related to Figure 4**

(A) Gene expression of *NOTO*, *TBXT*, *KRT8, KRT18, NOGGIN* and *SHH* during the monolayer and 3D pellet culture period. Significance indicated at day 28 CRISPRa transfected pellets compared with day 7 monolayer cells. Data presented as mean ± SD, **P<0.01, ***P<0.001, ****P<0.0001, ns: no significance, n=3 biological replicates (generated independently).

(B) Representative immunofluorescence staining of TBXT of day 28 NS9 and NTS9 pellets.
(C) Representative immunofluorescence staining of panKRT of day 28 NTS9 pellet.
(D) Representative immunofluorescence staining of ACAN and mScarlet of day 28 NS9 and NTS9 pellets.
(E and F) Representative H&E and Alcian blue staining images of the day 28 NS and NTS9 pellets showing a matrix-rich core.
Scalebars: 200 µm in top images and 100 µm in bottom images in B, C and D; 100 µm in left images and 40 µm in right images in E and F.
NS9: *NOTO*-g1+*SOX9*-g7; NTS9: *NOTO*-g1+*TBXT*-g3+*SOX9*-g7; SOX-trio: *SOX9*-g7+*SOX5*-g6+*SOX6*-g7

**Table S1: List of CRISPRa gRNA sequences, related to Figure 1 and S1**

| Genes | Guide sequence | Library |
|---|---|---|
| *NOTO*-g1 | GTGAGCGCCCGCTCTGTGCG | Sanson, K. R. et al. (2018) |
| *NOTO*-g2 | CCGCTCTGTGCGAGGCAAGG | Sanson, K. R. et al. (2018) |
| *NOTO*-g3 | CACCTTGCCTCGCACAGAGC | Sanson, K. R. et al. (2018) |
| *TBXT*-g1 | GAGTGTGCCAGGCGTGTGCG | Sanson, K. R. et al. (2018) |
| *TBXT*-g2 | CGGAAGGTGGGAGAAGGTCG | Sanson, K. R. et al. (2018) |
| *TBXT*-g3 | GTCATTGCTTTCACGGAAGG | Sanson, K. R. et al. (2018) |
| *FOXA2*-g1 | CTCTCGCGCTCCCTCTCCCT | Sanson, K. R. et al. (2018) |
| *FOXA2*-g2 | AGAGGGAGCGCGAGAGAGGG | Sanson, K. R. et al. (2018) |
| *FOXA2*-g3 | GGGAGGAGGGGACGGTGCTT | Sanson, K. R. et al. (2018) |
| *SOX9*-g1 | ATCACAGCACAGCAGTCCCA | Sanson, K. R. et al. (2018) |
| *SOX9*-g2 | CACTGGGCTGGGTGACGAGG | Sanson, K. R. et al. (2018) |
| *SOX9*-g3 | CAGCACAGCAGTCCCAGGGT | Sanson, K. R. et al. (2018) |
| *SOX9*-g4 | TGGCACTGGGCTGGGTGACG | Sanson, K. R. et al. (2018) |
| *SOX9*-g5 | ATCAGCTGCCTGCCAACCCT | Sanson, K. R. et al. (2018) |
| *SOX9*-g6 | CCTCCTCCCTCCCCAAAATC | Sanson, K. R. et al. (2018) |
| *SOX9*-g7 | AATCAGCTGCCTGCCAACCC | Konermann, S. et al. (2015) |
| *SOX9*-g8 | GATCGCAGCCAAAGGGCGGA | Konermann, S. et al. (2015) |
| *SOX5*-g1 | TAGCCCCCAGTTAATCACTT | Konermann, S. et al. (2015) |
| *SOX5*-g2 | TCAGTTGATTAGATTTTCTG | Konermann, S. et al. (2015) |
| *SOX5*-g3 | TCTGGAAATGGAGCAGACAA | Konermann, S. et al. (2015) |
| *SOX5*-g4 | CCTCGTCCTTGAGAGCAACA | Sanson, K. R. et al. (2018) |
| *SOX5*-g5 | TCCCTTTGCTGGTCATGAGG | Sanson, K. R. et al. (2018) |
| *SOX5*-g6 | TCGTCCTTGAGAGCAACACG | Sanson, K. R. et al. (2018) |
| *SOX5*-g7 | AAATCCCTTTGCTGGTCATG | Sanson, K. R. et al. (2018) |
| *SOX5*-g8 | CCGTGTTGCTCTCAAGGACG | Sanson, K. R. et al. (2018) |
| *SOX5*-g9 | GCCGCCTCATGACCAGCAAA | Sanson, K. R. et al. (2018) |
| *SOX6*-g1 | CCTCAGATCACCTTCAACTT | Konermann, S. et al. (2015) |
| *SOX6*-g2 | GCCTCTTGCACCAAAGTTGA | Konermann, S. et al. (2015) |
| *SOX6*-g3 | TGTCCTTTATAAAGGCAAAA | Konermann, S. et al. (2015) |
| *SOX6*-g4 | TCTTGCCTGTGTTGTCTGGG | Sanson, K. R. et al. (2018) |
| *SOX6*-g5 | GCTCCCCTCCCAGACAACAC | Sanson, K. R. et al. (2018) |
| *SOX6*-g6 | TTGCCTGTGTTGTCTGGGAG | Sanson, K. R. et al. (2018) |
| *SOX6*-g7 | CAAGATGAGACAAGAGGCGA | Sanson, K. R. et al. (2018) |
| *SOX6*-g8 | CTTGCCTGTGTTGTCTGGGA | Sanson, K. R. et al. (2018) |

**Table S2: Set up of the iNLC differentiation in this study, related to Figure 2-4 and S2-S4**

| Part of Study | Cell State | gRNAs | FACS | TGF-β1 | Note |
|---|---|---|---|---|---|
| Stage 1 (Broad screen) 7 days in monolayer culture | iPSCs → iMEPCs → CRISPRa guides sets | N, NT, NTF, NTS9, NS9, NTF+S9, NTF+SOX-trio, SOX-trio | No sorting | No | Detect initial lineage bias and select best-performing guides |
| Stage 2 (High-resolution lineage mapping) 7 days in monolayer culture | Same differentiation as in Stage 1 but with CRISPRa-transfected cells purification | NS9, NTS9, SOX-trio | FACS sorted for CRISPRa activation reporter | No | Define cluster identity and co-expression structure |
| Stage 3 (Maturation) 7 days in monolayer and 21 days in 3D pellet | Day-7 cultures aggregated into pellets | NS9, NTS9, SOX-trio | No sorting | With TGF-β1 in 3D pellet | Assess matrix-producing phenotype and maturation trajectory |

**Table S3: Cell distribution in clusters by plate after filtering, related to Figure 3 and S3**

| Cluster | NS9-1 | NS9-2 | **NS9 total** | NTS9-1 | NTS9-2 | **NTS9 total** | SOX-trio-1* | SOX-trio-2 | SOX-trio-3 | **SOX-trio total** |
|---|---|---|---|---|---|---|---|---|---|---|
| neuromesoderm | 111 | 145 | **256** | 6 | 1 | **7** | 5 | 22 | 20 | **47** |
| ratio | 79.9% | 88.4% | **84.5%** | 7.1% | 1.4% | **4.6%** | 7.8% | 9.7% | 9.2% | **9.3%** |
| mesoderm progenitor-1 | 10 | 5 | **15** | 35 | 26 | **61** | 21 | 61 | 62 | **144** |
| ratio | 7.2% | 3.0% | **5.0%** | 41.7% | 37.7% | **39.9%** | 32.8% | 27.0% | 28.6% | **28.4%** |
| mesoderm progenitor -2 | 7 | 4 | **11** | 6 | 18 | **24** | 13 | 72 | 68 | **153** |
| ratio | 5.0% | 2.4% | **3.6%** | 7.1% | 26.1% | **15.7%** | 20.3% | 31.9% | 31.3% | **30.2%** |
| mesoderm progenitor -3 | 9 | 7 | **16** | 34 | 24 | **58** | 11 | 38 | 40 | **89** |
| ratio | 6.5% | 4.3% | **5.3%** | 40.5% | 34.8% | **37.9%** | 17.2% | 16.8% | 18.4% | **17.6%** |
| notochord | 2 | 3 | **5** | 3 | 0 | **3** | 14 | 33 | 27 | **74** |
| ratio | 1.4% | 1.8% | **1.7%** | 3.6% | 0.0% | **2.0%** | 21.9% | 14.6% | 12.4% | **14.6%** |
| total | 139 | 164 | **303** | 84 | 69 | **153** | 64 | 226 | 217 | **507** |

Technical replicates were processed for each transcription factor combination sorted on separate plates for single cell sequencing. A biological replicate was conducted for SOX-trio. Note that for technical reasons data was not available from the first technical replicate of SOX-trio-1, and SOX-trio-2 and SOX-trio-3 are second technical replicate.

**Table S4: *mNeonGreen*-positive cells distribution after filtering, related to Figure 3 and S3**

| Conditions | neuro mesoderm | mesoderm progenitor -1 | mesoderm progenitor -2 | mesoderm progenitor -3 | notochord |
|---|---|---|---|---|---|
| Total cell number | 310 | 220 | 188 | 163 | 82 |
| *mNeonGreen+* | 12 | 30 | 40 | 16 | 44 |
| ratio | 3.9% | 13.6% | 21.3% | 9.8% | 53.7% |
| *mNeonGreen-* | 298 | 190 | 148 | 147 | 38 |
| ratio | 96.1% | 86.4% | 78.7% | 90.2% | 46.3% |

**Table S5: Medium preparation, related to Figure 2-3 and S2-S3**

| N2B27 MEDIUM | | | | |
|---|---|---|---|---|
| **COMPONENT** | **Supplier** | **Identifier** | **Stock concentration** | **Final concentration** |
| DMEM/F-12 | Gibco | 31331028 | - | - |
| NEAA | Gibco | 11140050 | 100X | 1X |
| GLUTAMAX | Gibco | 35050061 | 100X | 1X |
| BETA-MERCAPTOETHANOL | Gibco | 31350010 | 50 mM | 0.001 |
| N2 SUPPLEMENT | Gibco | 17502048 | 100X | 0.5X |
| B27 SUPPLEMENT | Gibco | 17504044 | 50X | 0.5X |
| BOVINE ALBUMIN | Sigma | A1595 | 0.1 | 0.00005 |

**Table S6: List of qPCR primers, related to Figure 1, 2, 4, S1, S2 and S4**

| **Genes** | **Forward sequence** | **Reverse sequence** | **Tm (°C)** |
|---|---|---|---|
| *NANOG* | CCAGAGAATGAAATCTAAGAGGTG | CAGGTTGAATTGTTCCAGGT | 60 |
| *OCT4* | GAGAACCGAGTGAGAGGCAACC | CATAGTCGCTGCTTGATCGCTTG | 60 |
| *NODAL* | TGGAAGGCTGGGAAACTC | CTCCTCTTCAGTTCTGGTGG | 57 |
| *LEFTY1* | CATCGAGGGACTTGACTTGTG | AGAGCATTTGTCCATCAGCAG | 63.5 |
| *SOX17* | TCTGCCTCCTCCACGAAG | CAGATTCCAGACCTGCACAA | 60 |
| *TBX6* | CCTGTGTCTTTCCATCGTGTC | GCCCGAACTAGGTGTATGC | 58 |
| *NOTO* | AAAGCCTCCTCAACCAGAAG | GAGTCTGTGCCTCCAATCAT | 60 |
| *TBXT* | CCTGAGACCCAGTTCATAGC | GTTCCTCCATCATCTCTTTGTG | 64 |
| *FOXA2* | TACGTGTTCATGCCGTTCAT | CGACTGGAGCAGCTACTATGC | 64 |
| *SOX9* | CAAGCTCTGGAGACTTCTGAACG | CCGTTCTTCACCGACTTCCT | 60 |
| *SOX5* | CCTCAAAGCCTCTGTCCCAG | TGCCTTGGTGACAGCATCAT | 60 |
| *SOX6* | AACAACGGCAGCAAATGGAC | TGGATCTGTTGCTGCAGGAG | 60 |
| *NOGGIN* | GGAGGAAGTTACAGATGTGGCTGT | CACTCGGAAATGATGGGGTACTG | 63.5 |
| *SHH* | GCGATTTAAGGAACTCACCC | ACTTGTCCTTACACCTCTGAG | 61.5 |
| *COL2A1* | AGGGCCAGGATGTCCGGCA | GGGTCCCAGGTTCTCCATCT | 63 |
| *ACAN* | CAACTACCCGGCCATCC | GATGGCTCTGTAATGGAACAC | 64 |
| *mScarlet* | CACCAACTTCCCTCCTGACG | GGTGTAGTCCTCGTTGTGGG | 60 |
| *GAPDH* | CAAGATCATCAGCAATGCCT | CAGGGATGATGTTCTGGAGAG | 60 |
| *YWHAZ* | ACTTTTGGTACATTGTGGCTTCAA | CCGCCAGGACAAACCAGTAT | 64 |

Data S1. Differential expression gene list, related to Figure 3 and Figure S3. (Excel file)
