# Supplementary material for: CRISPR activation reveals SOX5/6/9 as key transcriptional regulators directing iPSC-derived cells toward a notochordal lineage
Source: Stem Cell Reports. 2026 Aug 27;21(9):103060. doi: 10.1016/j.stemcr.2026.103060 (PMC13555567; doi:10.1016/j.stemcr.2026.103060)
Supplement: Supplementary file 2 [file mmc3.pdf]

STEM CELL REPORTS
ISSCR
Resource
OPEN ACCESS

CRISPR activation reveals *SOX5/6/9* as key transcriptional regulators directing iPSC-derived cells toward a notochordal lineage

Xiaole Tong,[1,2] Marieke Visscher,[3] Frank M. Riemers,[1,2] Danielle Versluis,[1,2] Niels Geijsen,[4,5] Peng Shang,[4,5] Marianna A. Tryfonidou,[1,2,*] and Deepani W. Poramba-Liyanage[1,2,6,*]
[1]Department of Clinical Sciences, Faculty of Veterinary Medicine, Utrecht University, Yalelaan 108, 3584 CM Utrecht, the Netherlands
[2]Regenerative Medicine Center Utrecht, Uppsalalaan 8, 3584 CT Utrecht, the Netherlands
[3]NTrans Technologies BV, J. H. Oortweg 19, 2333 CH Leiden, the Netherlands
[4]Department of Anatomy and Embryology, Leiden University Medical Center, Einthovenweg 20, 2300 RC Leiden, the Netherlands
[5]The Novo Nordisk Foundation Center for Stem Cell Medicine (reNEW), Leiden node, Leiden, the Netherlands
[6]Lead contact
*Correspondence: m.a.tryfonidou@uu.nl (M.A.T.), deepsliyanage@gmail.com (D.W.P.-L.)
https://doi.org/10.1016/j.stemcr.2026.103060

SUMMARY

Intervertebral disc (IVD) degeneration, a leading cause of chronic lower back pain, is associated with loss of vacuolated notochordal cells (NCs) and fibrotic remodeling of the nucleus pulposus. Emerging therapies increasingly focus on NCs, which are rare but therapeutically relevant cells for regenerating degenerated IVDs. In this study, we used CRISPR-based transactivation (CRISPRa) to direct the differentiation of human induced pluripotent stem cells (iPSCs) into the NC lineage. We tested CRISPRa-mediated activation of *NOTO*, *TBXT*, *FOXA2*, *SOX5*, *SOX6*, and *SOX9*, coupled with single-cell sequencing of Aggrecan-2A-mScarlet reporter iPSCs. This approach identified the *SOX5/6/9* combination (SOX-trio) as critical for promoting NC lineage commitment. The SOX-trio yielded the largest cell population expressing a range of genes previously associated with NC identity, including *SHH*, *FOXA1*, *FOXA2*, *FOXJ1*, *FN1*, *ALCAM*, *KRT8*, and *KRT18*. Our study demonstrates the integration of CRISPRa with single-cell technologies as a powerful platform for investigating and enriching iPSC-derived NCs, supporting future regenerative strategies across various fields.

INTRODUCTION

Lower back pain (LBP) is a leading cause of disability worldwide (Wu et al., 2020). Intervertebral disc (IVD) degeneration, widely recognized as a major contributor to LBP, arises from structural and cellular changes within the core of the IVD, the nucleus pulposus (Muhl et al., 2020), particularly the loss of notochordal cells (NCs) (Luoma et al., 2000; Zhao et al., 2007; Cheung et al., 2012; Diwan and Melrose 2023). During early development, NCs maintain the proteoglycan-rich, hydrated extracellular matrix (ECM) of the NP through the production of key ECM components like aggrecan and collagen II. With aging, NCs are replaced by chondrocyte-like cells, which diminishes the healthy ECM maintenance and promotes degeneration (Hunter et al., 2004). Within this context, NCs have raised significant interest in regenerative therapies aimed at halting or reversing the IVD degeneration process (Bach et al., 2021).

Autologous NC treatment is impractical due to the scarcity of NCs in adult human IVDs, and harvesting cells through partial nuclectomy risks inducing degeneration in otherwise healthy discs (Alini et al., 2008). Transplanting allogeneic fetal or juvenile NCs poses ethical challenges, while xenogeneic approaches raise safety concerns (Bach et al., 2021). Induced pluripotent stem cells (iPSCs) offer a promising alternative, as they can be derived from any somatic cell type and possess high self-renewal and differentiation potential. By generating NC-like cells (NLCs), iPSCs circumvent the ethical concerns associated with allogeneic and xenogeneic NC transplantation (Revilla et al., 2016). Several studies have reported the iPSC to NLC (iNLC) differentiation by targeting essential transcription factor (TF) networks using growth factor supplementation or single key TF-encoding mRNA transfection (Tang et al., 2018; Colombier et al., 2020; Zhang et al., 2020; Warin et al., 2024). However, these approaches face challenges such as low differentiation efficiency and inherent cellular heterogeneity, limiting their therapeutic applicability.

The discovery that overexpression of just four TFs—*OCT3/4*, *SOX2*, *c-MYC*, and *KLF4*—could reprogram fibroblasts into iPSCs (Takahashi and Yamanaka 2006), has led to efforts using forced TF expression to drive the iPSC differentiation (Ng et al., 2021). In notochord development, *Brachyury* (*TBXT*) and *Forkhead box protein A2* (*FOXA2*) are pivotal TFs (Yamanaka et al., 2007). *TBXT* is critical for NC maturation while *FOXA2* is involved in the early notochord specification and together with *TBXT*, regulates the expression of downstream genes such as *NOTO* (Yamanaka et al., 2007). During notochord maturation, the lineage maintains a recognizable notochordal transcriptional memory, with *TBXT* and *FOXA2* remaining functionally relevant. Colombier et al. (2020) explored this, independently transfecting *NOTO*, *TBXT*, and *FOXA2* mRNA into human iPSC-derived mesendoderm progenitor cells (iMEPCs) to promote NC lineage commitment. The iMEPCs initially exhibited

Published by Elsevier Inc. on behalf of International Society for Stem Cell Research.
This is an open access article under the CC BY-NC-ND license (http://creativecommons.org/licenses/by-nc-nd/4.0/).

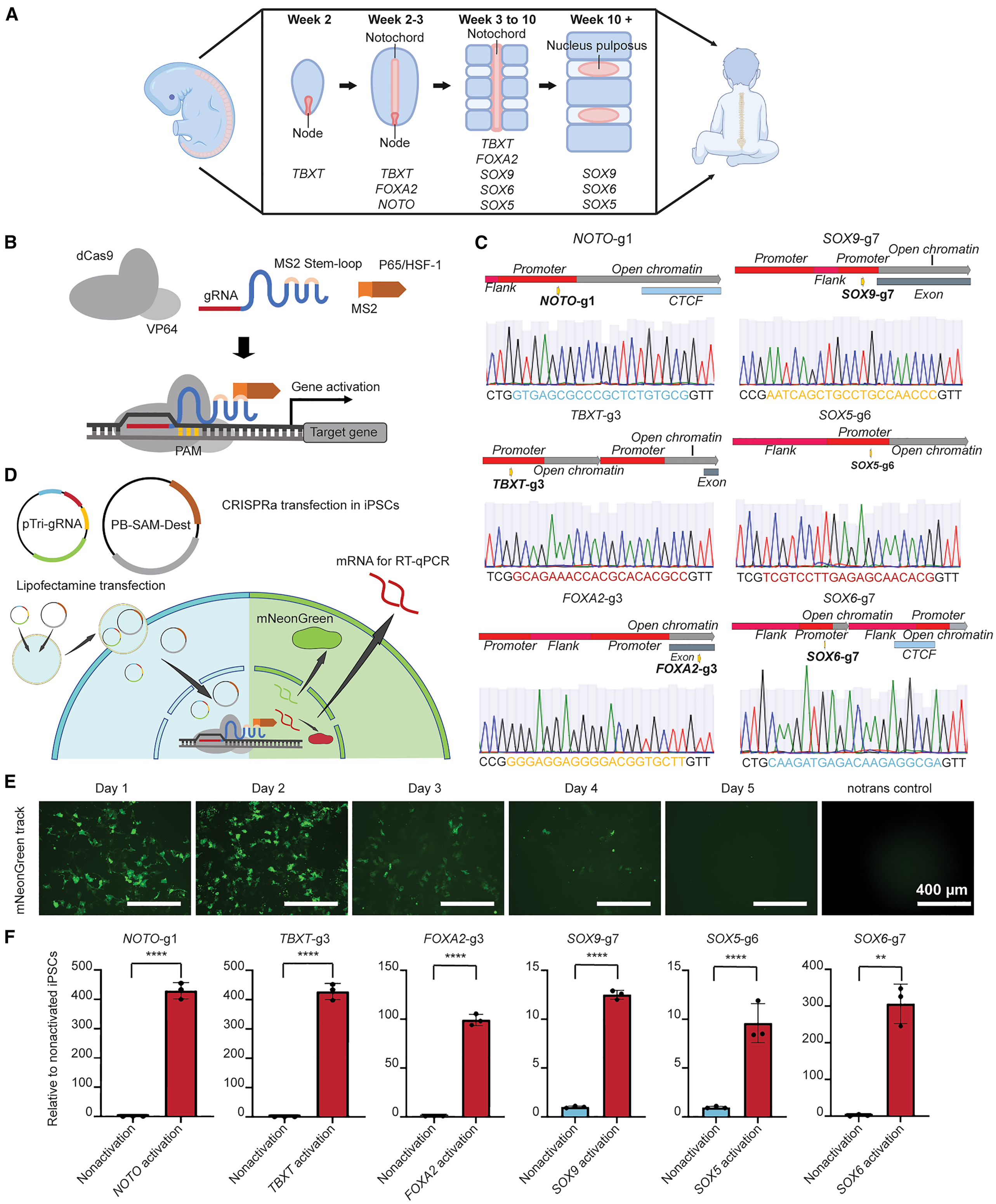

Figure 1. CRISPRa-mediated induction of target genes in iPSCs

(A) A schematic highlights the key genes in notochord and IVD development.

(B) Schematic of the CRISPRa-SAM system.

(legend continued on next page)

high TBXT and FOXA2 expression, which declined sharply with subsequent culture. mRNA transfection partly rescued the expression of these TFs, with *NOTO* transfection providing most effective NC population, yielding 6.6% of iNLCs (Colombier et al., 2020). A follow-up study using single-cell sequencing showed 23.8% of cells forming an iNLC cluster expressing *TBXT*, *FOXA2*, and *SOX9* (Warin et al., 2024). The *SOX9* induction in iNLCs suggests that *SOX9*, together with *NOTO*, has a central regulatory role in the NC lineage (Colombier et al., 2020; Warin et al., 2024). The *SOX* family, particularly *SOX5*, *SOX6*, and *SOX9* also play a significant role in notochord development. These *SOX* genes are dispensable for notochord formation but required for notochord maintenance. They are upregulated in coordination with SHH signaling during notochord maturation and are instrumental in regulating the expression of ECM components critical for the notochord's mechanical properties (Smits and Lefebvre 2003; Barrionuevo et al., 2006). Despite their known roles in notochord development, the potential of these *SOX* genes in guiding iNLC differentiation remains underexplored.

In this study, we established a proof-of-concept pipeline for CRSIRPa-mediated transcriptional modulation interrogating early transcriptional responses during lineage specification, which can be used for a larger scale TF screening to further improve the iNLC differentiation protocol. We tried to explore the minimal TF combinations to drive the iNLC differentiation by using ACAN-2A-mScarlet reporter human iPSC line (Tong et al., 2024). This iPSC reporter line can be differentiated into matrix-producing cell types, report for ACAN expression throughout the differentiation process, and hence represents a significant technical advancement toward optimizing iNLC differentiation. We implement CRISPRa-mediated transcriptional regulation to direct the iNLC differentiation. The CRISPRa strategy enables precise, physiologically relevant upregulation of multiple genes within the native genomic context (Maeder et al., 2013), which offers a powerful platform for investigating complex gene regulatory networks in iPSC differentiation. By testing CRISPR activation of *NOTO*, *TBXT*, *FOXA2*, *SOX5*, *SOX6*, and *SOX9*, combined with cell sorting and single-cell sequencing, we identified the SOX-trio (*SOX5*, *SOX6*, and *SOX9*) as pivotal factors for promoting NC lineage commitment.

RESULTS

Inducing target gene expression in iPSCs by the CRISPRa-SAM system

To optimize NC lineage commitment of iPSCs, we employed the CRISPRa-SAM system (Konermann et al., 2015) in TMOi001-A iPSCs to activate transcription of six key TFs, *NOTO*, *TBXT*, *FOXA2*, *SOX9*, *SOX5*, and *SOX6*, which are crucial to mammalian notochord formation and IVD development (Figure 1A) (Smits and Lefebvre 2003; Yamanaka et al., 2007; Warin et al., 2024). The CRISPRa transactivator dCas9-VP64 works synergistically with MS2-P65-HSF1 to achieve robust transcriptional activation, forming the synergistic activation mediator (SAM) system (Figures 1B and S1A) (Konermann et al., 2015).

To activate the six TF genes, each one was targeted with 3–9 previously identified gRNAs, which are effective in CRISPR-Cas9-based gene editing (Figures 1C and S1B; Table S1) (Konermann et al., 2015; Sanson et al., 2018). Two days post-electroporation, RT-qPCR revealed augmented target gene expression by 1.5 to 100-fold, while some gRNAs failed to induce activation (Figures S1C and S1D). The most effective gRNAs were further validated using lipofectamine transfection, a gentler transfection method also used for iPSC differentiation (Colombier et al., 2020; Nakami et al., 2022; Warin et al., 2024). The *mNeonGreen* marker in pTri-gRNA served as a transfection indicator and was preliminarily validated. The mNeonGreen expression was visible up to 5 days after transfection (Figures 1D and 1E). The retention of CRISPRa plasmids was further evaluated by flow cytometry during the subsequent iNLC differentiation process. Significant gene activation was confirmed with the transfection of the CRISPRa-SAM system, while no significant induction was detectable in all nonactivated control iPSCs (Figure 1F). Based on these results, the most potent gRNAs were selected for further experiments: guide 1 (g1) for *NOTO*; g3 for *TBXT*; g3 for *FOXA2*; g7 for *SOX9*; g6 for *SOX5*; and g7 for *SOX6* (Figure 1C) to assess the role of targeted TF activation in NC lineage commitment.

CRISPRa-mediated induction of target genes promotes NC lineage commitment of iPSCs

To test if CRISPRa-mediated induction of key TFs promotes NC lineage commitment of iPSCs, six gRNAs combinations

(C) Genome browser view of the respective TF loci in the GRCh38 human genome with the selected gRNAs. Sanger sequencing shows the correct integration of gRNA sequences and plasmid backbone.
(D) gRNA selection pipeline. CRISPRa-SAM plasmids were transfected into human TMOi001-A iPSCs using lipofectamine, and cells were collected after 3 days for RT-qPCR.
(E) mNeonGreen expression coupled to the pTri-gRNA plasmid transfection. Scale bars, 400 μm.
(F) RT-qPCR showing activation of target genes by the top-performing gRNAs. Data are presented as mean ± SD; $^{**}p < 0.01$, $^{**}p < 0.0001$, ns = not significant; $n = 3$ biological replicates (independent transfections). Validation of all gRNAs is shown in Figure S1.

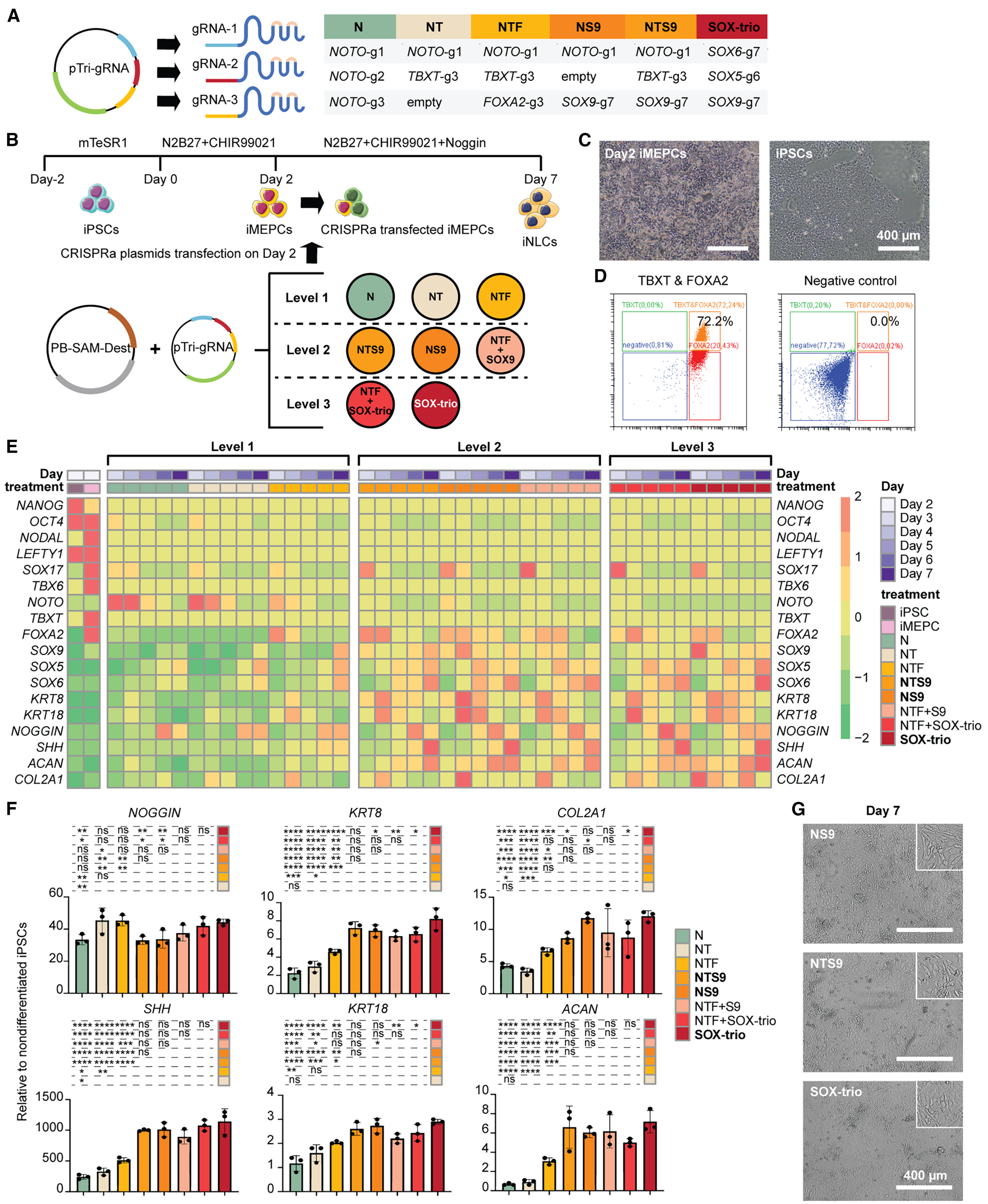

(legend on next page)

were designed, targeting selected candidate TFs (Figure 2A; Table S2). These combinations represent a focused, hypothesis-driven subset informed by developmental evidence, rather than an exhaustive combinatorial screen, enabling systematic testing of regulators of notochord initiation (*NOTO*, *TBXT*, and *FOXA2*) and maturation (*SOX9*, *SOX5*, and *SOX6*) (Smits and Lefebvre 2003; Yamanaka et al., 2007; Warin et al., 2024). Specifically, we first included *NOTO* guides (N), *NOTO*-g1+*TBXT*-g3 (NT) and *NOTO*-g1+*TBXT*-g3+*FOXA2*-g3 (NTF) (Figure 2A), based on studies in mice that highlight *TBXT* and *FOXA2* as key regulators of the NC lineage onset (Yamanaka et al., 2007). Furthermore, Colombier et al. (2020) demonstrated that single *NOTO* mRNA transfection in iMEPCs maintained TBXT and FOXA2 expression yielding iNLCs. This approach faced variability in cell fate outcomes, with a maximum yield of 6.6% iNLCs (Colombier et al., 2020). To address these limitations, *SOX9* was added due to its role in preserving the notochord structure (Barrionuevo et al., 2006). However, *SOX9* is an important regulator of chondrogenesis (Lefebvre and Dvir-Ginzberg 2017), and hence sole transactivation can direct iPSCs differentiating into the chondrocytes. To mitigate this, *SOX9* was included in the combinations: *NOTO*-g1+*SOX9*-g7 (NS9), *NOTO*-g1+*TBXT*-g3+*SOX9*-g7 (NTS9), and NTF+*SOX9*-g7 (NTF+S9) (Figure 2A). Finally, combinations incorporating *SOX5* and *SOX6* were included, *SOX9*-g7+*SOX5*-g6+*SOX6*-g7 (SOX-trio) and NTF+SOX-trio (Figure 2A), given their cooperative roles with *SOX9* and importance in ECM regulation contributing to NC survival and maintenance (Han and Lefebvre 2008).

To assess each combination's effect on iNLC differentiation, TMOi001-A iPSC line was first driven into iMEPCs (Tong et al., 2024), and followed by CRISPRa transfection (Figure 2B; Table S2) (Warin et al., 2024). The CRISPRa transfection in iMEPCs was tracked by mNeonGreen expression (Figure S2A). Morphological features were observed during differentiation, with cells progressively losing their typical iPSC morphology, characterized by tightly packed cells arranged in rounded colonies (Figure 2C).

Upon exposure to WNT activation, more than 70% of the cells co-expressed TBXT and FOXA2 by day 2 (Figures 2C, 2D, S2B and S2C), while pluripotency markers consistently decreased over time (Figures 2E and S2D). Highly expressed *NODAL* and *LEFTY1* on day 2 indicated successful WNT activation, whereafter they decreased in all conditions (Figures 2E and S2D). *SOX17* and *TBX6*, typical endoderm and mesoderm markers, respectively, also gradually decreased after the CRISPRa transfection (Figures 2E and S2D). In all situations, CRISPRa induction led to a sharp and significant increase in the expression of the respective activated TF 24 h post-transfection, with N achieving the highest *NOTO* expression and NTF yielding the highest *TBXT* and *FOXA2* levels (Figures 2E and S2E). SOX-trio resulted in the highest upregulation of *SOX9* and *SOX6*, while *SOX5* was comparable among the different combinations except for N, NT, and NTF (Figures 2E and S2E).

We next sought to determine the minimal TF combination required to promote NC lineage commitment. We focused on the expression of a key gene combination in NC lineage commitment, *NOGGIN*, *SHH*, *KRT8*, *KRT18*, *COL2A1*, and *ACAN* at the end of the 7-day differentiation as an early NC predictor (Warin et al., 2024). Other typical NC markers such as *NOTO* and *TBXT* were excluded from this panel, as they were directly targeted by CRISPRa. The analysis was conducted in three levels: (Sive et al., 2002) by (1) evaluating the effects of the three TFs studied in Colombier et al. (2020) (Colombier et al., 2020); followed by (2) the additive value of *SOX9*, and (3) ultimately evaluating the effects of the SOX-trio combination. N, NT, and NTF were compared first (Figure 2B; level 1). The results on day 7 demonstrate that the NTF resulted in significantly higher expression of *SHH*, *KRT8*, *COL2A1*, and *ACAN* compared to N and NT, underscoring the positive impact of this combination on NC lineage commitment (Figure 2F). Both NT and NTF showed significantly higher expression of *NOGGIN* compared to N (Figure 2F), suggesting that *TBXT* and *FOXA2* contribute to early differentiation. The inclusion of the *SOX9* gene (NS9, NTS9, and

Figure 2. CRISPRa-mediated induction of target genes to promote NC lineage commitment

(A) The overview of different gRNA combinations.

(B) The process of CRISPRa-mediated iNLC differentiation. TMOi001-A iPSCs were driven into iMEPCs, transfected with CRISPRa plasmids at day 2, and collected daily for 5 days for RT-qPCR.

(C) Microscopical images show the typical iPSCs and iMEPCs morphology on day 2 prior to CRISPRa transfection.

(D) FACS outcomes in day 2 iMEPCs.

(E) Heatmap of relative gene expressions at different time points after transfection.

(F) Corresponding day 7 histograms of key NC markers. Significance indicates differences between the gRNA combinations used.

(G) Cell morphology of day 7 differentiated cells in NS9, NTS9, and SOX-trio groups.

Scale bars: 400 μm in (C) and (G).

N: *NOTO* guides; NT: *NOTO*-g1+*TBXT*-g3; NTF: *NOTO*-g1+*TBXT*-g3+*FOXA2*-g3; NS9: *NOTO*-g1+*SOX9*-g7; NTS9: *NOTO*-g1+*TBXT*-g3+*SOX9*-g7; NTF+S9: *NOTO*-g1+*TBXT*-g3+*FOXA2*-g3+*SOX9*-g7; SOX-trio: *SOX9*-g7+*SOX5*-g6+*SOX6*-g7; NTF+SOX-trio: *NOTO*-g1+*TBXT*-g3+*FOXA2*-g3+ *SOX9*-g7+*SOX5*-g6+*SOX6*-g7

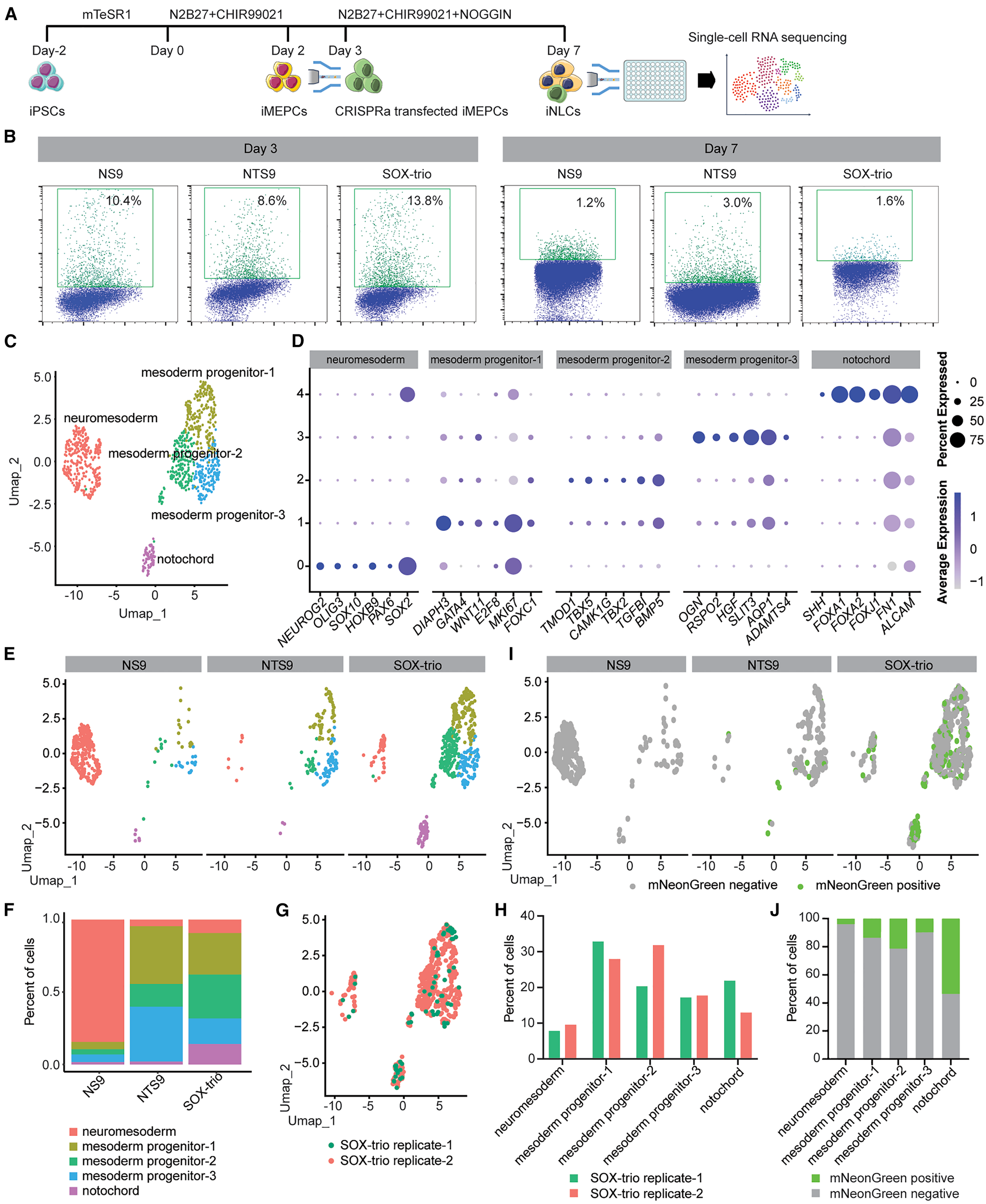

(legend on next page)

NTF+S9) (Figure 2B; level 2) further augmented the expression of *SHH*, *KRT8*, *COL2A1*, and *ACAN* compared to their respective counterparts, i.e., N and NTF, confirming the critical role of *SOX9* in notochord development. However, *SOX9* had no significant impact on *NOGGIN* expression (Figure 2F). Finally, the inclusion of *SOX5* and *SOX6* within the SOX-trio (NTF+SOX-trio) (Figure 2B; level 3) did not significantly enhance the expression of the key NC markers compared to NTF+S9 (Figure 2F). Nonetheless, *SHH*, *KRT8*, and *ACAN* levels were significantly higher in NTF+SOX-trio than in NTF alone (Figure 2F), demonstrating added benefit of including *SOX5* and *SOX6*. Furthermore, SOX-trio, as a minimal set of TFs, performed similarly to the more extensive NTF+SOX-trio, underscoring its efficiency in directing NC lineage commitment. Interestingly, neither NTF+S9 nor NTF+SOX-trio exhibited a synergistic effect when all TFs were activated together (Figure 2F).

Seeing the beneficial effects of *SOX* genes and striving for a minimal TF combination contributing to iNLC differentiation, NS9, NTS9, and SOX-trio were selected for follow-up. A time course analysis from day 2 to day 7 was conducted to evaluate the overall effect of NS9, NTS9, and SOX-trio. The morphological changes indicate progression of differentiation, with no evident cell death or overconfluence observed and no clear microscopic differences among the three conditions (Figures 2G and S2F). The key genes in the NC lineage *NOGGIN*, *SHH*, and *ACAN* increased gradually in the non-transfected cells reaching significance on day 7 but remained at much lower levels compared to the transactivated groups (Figure S2G). In the three transactivated groups, peak expression of *NOGGIN* was reached on day 6, while peak expression levels of *SHH* and *ACAN* were observed at the end of the culture period. Interestingly, *COL2A1* levels remained stable throughout the differentiation process but were still significantly higher on day 7 compared to day 2 for all groups (Figure S2G). Based on these results, NS9, NTS9, and SOX-trio were confirmed as minimal gene combinations that significantly promoted iNLC differentiation and were selected for further analysis.

Single-cell RNA sequencing highlights the key contribution of *SOX* genes to NC-NPC lineage commitment

To better understand the heterogeneity of CRISPRa-mediated iNLC differentiation, we examined the effects of the three minimal gene combinations—NS9, NTS9, and SOX-trio—eliciting the most outspoken effects. Sort sequencing (SORT-seq) was performed on the ACAN-2A-mScarlet reporter iPSCs (TMOi001-A-14 line) following CRISPRa-SAM induction (Table S2; Figure S3A) (Tong et al., 2024). mNeonGreen-positive cells were sorted 24 h post-transfection, yielding 8%–13% *mNeonGreen*-positive cells (Figures 3A and 3B). These sorted cells were then differentiated into the NC lineage, and SORT-seq was performed in day 7 differentiated iPSCs (Muraro et al., 2016). Additionally, CRISPRa plasmids retention was assessed in day 7 cells by detecting of *mNeonGreen* expression on flow cytometry, and 1%–3% *mNeonGreen*-positive cells were observed (Figures 3A and 3B).

SORT-seq retrieved 2,688 cells grouped into five distinct clusters (Figures 3C, 3D, and S3B; Data S1). Based on known annotation markers, these clusters were classified as notochord (*SHH*, *FOXA1*, *FOXA2*, *FOXJ1*, *FN1*, and *ALCAM*) (Warin et al., 2024), mesoderm progenitor-1 (*DIAPH3*, *GATA4*, *WNT11*, *E2F8*, *MKI67*, and *FOXC1*), mesoderm progenitor-2 (*TMOD1*, *TBX5*, *CAMK1G*, *TBX2*, *TGFBI*, and *BMP5*), mesoderm progenitor-3 (*OGN*, *RSPO2*, *HGF*, *SLIT3*, *AQP1*, and *ADAMTS4*), and neuromesoderm (*NEUROG2*, *OLIG3*, *SOX10*, *HOXB9*, *PAX6*, and *SOX2*) (Figures 3C, 3D, and S3B) (Nelms and Labosky 2010). Additionally, expression of *KRT8* and *KRT18* was detected in the notochord cluster, whereas *RUNX2* expression was undetectable (Figure S3C). The presence of mesoderm progenitor and neuromesoderm clusters confirms lineage heterogeneity. However, the emergence of a distinct notochord cluster demonstrates successful bias toward a notochordal trajectory consistent with our BMP-inhibition and TF-activation strategy.

Figure 3. Single-cell RNA sequencing highlights the key contribution of the SOX-trio in NC lineage commitment

(A) Schematic of the CRISPRa-mediated iNLC differentiation with mNeonGreen-positive cells sorting performed 24 h post-transfection and SORT-seq performed in day 7 differentiated iPSCs.

(B) FACS plots illustrating the percentage of mNeonGreen-positive cells on day 3 and day 7.

(C and D) The UMAP-based analysis visualized five clusters. Respective cell numbers and their proportions relative to the total cell number in each condition are shown in Table S3.

(E) UMAP-based plots indicate the contribution of each TF combination to the five clusters.

(F) Percentage of cells contributing to each cluster in different TF combinations.

(G and H) UMAP-based plots indicate the comparable distribution of the biological replicate of SOX-trio transactivations.

(I) The distribution of *mNeonGreen*-positive cells after filtering. Note that in NS9 and NTS9, fewer *mNeonGreen*-positive cells survived the filtration steps, indicating lower cell viability.

(J) Percentage of *mNeonGreen*-positive and -negative cells in each cluster for the three TF combinations.

NS9: *NOTO*-g1+*SOX9*-g7; NTS9: *NOTO*-g1+*TBXT*-g3+*SOX9*-g7; SOX-trio: *SOX9*-g7+*SOX5*-g6+*SOX6*-g7

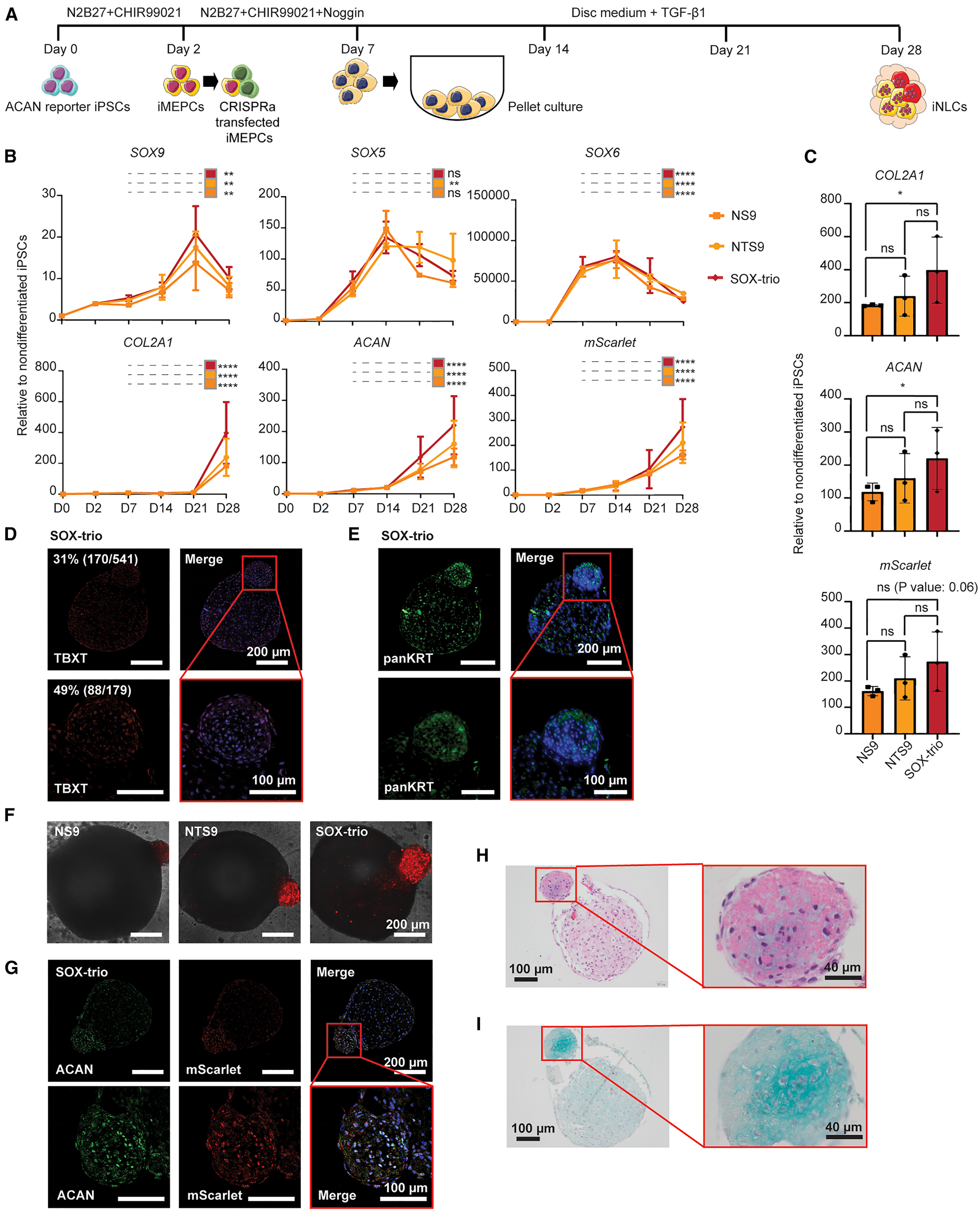

(legend on next page)

Among the TF combinations tested, SOX-trio (*SOX5/6/9*) activation yielded by far the largest notochord cluster, contributing 14.6% of the total cell population, compared with 1.7% and 2.0% under the NS9 and NTS9 conditions, respectively (Figures 3E, 3F, and S3D–S3F; Table S3). This enrichment was reproducible across biological replicates (13.5%–21.9% of cells; Figures 3G and 3H; Table S3). Importantly, over 90% of all cells in the notochord cluster originated from SOX-trio activated iMEPCs, confirming that coordinated activation of *SOX5*, *SOX6*, and *SOX9* is the dominant driver of notochordal differentiation in this system. This is consistent with the established role of *SOX9* as a central regulator of notochordal and chondrogenic gene networks (Figures 3F, S3E, and S3F) (Smits and Lefebvre 2003; Warin et al., 2024).

In contrast, the NS9 and NTS9 combinations contributed considerably fewer cells to the notochord cluster (Figures 3F, S3E, and S3F). To understand this discrepancy, we examined whether it could stem from variations in CRISPRa plasmid retention, potentially influencing transcriptional activation. mNeonGreen expression serves as a readout for both transfection efficiency and post-transfection analysis. Using SORT-seq, we analyzed whether plasmid retention enhanced CRISPR activation or reflected a state of early differentiation—where cells have reduced or ceased proliferation—by examining the distribution of *mNeonGreen*-positive cells across clusters at day 7 (Figures 3I, 3J, and S3G). Approximately 31% of the *mNeonGreen*-positive population was identified within the notochord cluster (Table S4) and 53.7% of notochord cluster cells retained *mNeonGreen* signal (Figure 3J; Table S4). In contrast, only 3.9% of the neuromesoderm cluster and 13.6%, 21.3% and 9.8% of the three mesoderm progenitor clusters, were *mNeonGreen*-positive (Figure 3J; Table S4).

Cells with lower *mNeonGreen* signal likely underwent additional divisions, whereas sustained fluorescence indicated reduced proliferation, typical of early differentiation. Thus, the persistence of *mNeonGreen* positivity in the notochord cluster reflects early notochord development and reduced proliferation. This is consistent with the known behavior of early notochordal progenitors, which diverge from bipotent neuromesoderm progenitors and contribute to midline axial structures in vertebrate embryos (Semprich et al., 2022). As expected for this stage of monolyer differentiation, none of the *mNeonGreen*-positive cells showed *mScarlet*, indicating that ACAN production has not yet initiated (Tong et al., 2024).

Matrix deposition during 3D pellet culture by iPSCs directed to the NC lineage through CRISPRa

Building on our findings that CRISPRa-mediated differentiation enhances NC lineage commitment, we next sought to assess how these cells mature in a physiologically relevant three-dimensional (3D) environment. Culturing cells in a 3D microenvironment is known to influence matrix deposition and has been previously applied in NC lineage differentiation (Tang et al., 2018; Tong et al., 2024). Therefore, following the monolayer differentiation, a 3D pellet culture system was established to further mature CRISPRa-driven differentiated iPSCs (Figure 4A; Table S2). This approach was applied to the three selected minimal gene combinations: NS9, NTS9, and SOX-trio using the ACAN-2A-mScarlet reporter line to track the matrix production (Table S2).

The expression of the *SOX* genes increased during 3D maturation, peaking at different time points (*SOX9* on day 21, and *SOX5* and *SOX6* on day 14) before declining in all three transactivated groups (Figure 4B). By day 28, *SOX9* and *SOX6* remained significantly higher than day 7 monolayer cultures across conditions, while *SOX5* was significantly elevated in the NTS9 group (Figure 4B). In contrast, *NOTO* expression declined from day 7 onward, with significantly lower levels at day 28 in the NS9 and

Figure 4. Matrix deposition by iPSCs promoted to the NC lineage through CRISPRa during 3D pellet culture

(A) The process of iNLC differentiation includes 3D pellet culture. Day 7 monolayer differentiating cells were pelleted and cultured in disc medium with TGF-β1 for 21 days.

(B) Gene expression of *SOX* genes, NC maturation genes and *mScarlet* reporter gene. Significance indicated at day 28 pellets compared with day 7 monolayer cells.

(C) Gene expression of the maturation genes and the *mScarlet* reporter gene in day 28 pellets. Data presented as mean ± SD, $^{**}p < 0.01$, $^{****}p < 0.0001$, ns: no significance, $n = 3$ biological replicates (generated independently).

(D) Representative immunofluorescence staining of TBXT of day 28 SOX-trio pellet.

(E) Representative immunofluorescence staining of panKRT of day 28 SOX-trio pellet.

(F) Fluorescent live cell imaging of day 28 pellets exhibiting characteristic morphology in which matrix-producing cells form a spatially enriched subregion.

(G) Representative immunofluorescence staining of ACAN and mScarlet of day 28 SOX-trio pellet.

(H and I) H&E and alcian blue staining of the day 28 SOX-trio pellet.

Scale bars: 200 μm in top images and 100 μm in bottom images in (D), (E) and (G); 200 μm in (F). 100 μm in left images and 40 μm in right images in (H) and (I).

NS9: *NOTO*-g1+*SOX9*-g7; NTS9: *NOTO*-g1+*TBXT*-g3+*SOX9*-g7; SOX-trio: *SOX9*-g7+*SOX5*-g6+*SOX6*-g7

NTS9 groups (Figure S4A). *TBXT* showed an early peak at the iMEPC stage (day 2) but sharply decreased after the CRISPRa transfection in all groups (Figure S4A).

We next examined *NOGGIN* and *SHH*, key genes expressed by the early notochord (Choi and Harfe 2011; Fausett et al., 2014). Unlike *NOTO* and *TBXT*, the expression of *NOGGIN* progressively increased, reaching significantly higher levels by day 28 pellets across all three groups. Peak expression of *SHH* was reached at day 7 cells but declined sharply during 3D culture (Figure S4A). ECM markers including *COL2A1* and *ACAN* and the *mScarlet* reporter also increased during the 3D differentiation. By day 28, all three groups showed significantly higher expression compared to day 7 monolayer differentiated cells, confirming the positive effect of 3D culture on NC maturation (Figure 4B). Notably, on day 28, the SOX-trio condition exhibited significantly higher expression of *COL2A1*, *ACAN*, and *mScarlet* compared to NS9 and NTS9, confirming the role of *SOX* genes in promoting ECM synthesis (Figure 4C).

To further assess cell phenotype, matrix synthesis and deposition, the day 28 pellets were assessed with fluorescent live cell imaging and immunofluorescence staining. TBXT was expressed in 29%–37% of cells within each tissue section, implying the presence of iNLCs across all conditions (Figures 4D and S4B). In addition, positive panKRT staining was observed in the SOX-trio and NTS9 groups. The staining in NS9 group could not be evaluated due to a technical issue during the staining procedure (Figures 4E and S4C). Consistent with the RT-qPCR data, a small but distinct mScarlet-positive pellet region was observed in all three groups (Figure 4F), further corroborated by ACAN and mScarlet immunopositivity (Figures 4G and S4D). Histology stainings revealed early matrix deposition hubs, characterized by matrix-rich regions that overlapped with the ACAN and mScarlet positive areas (Figures 4G–4I and S4D–S4F). Interestingly, the SOX-trio and NTS9 groups exhibited a higher ratio of TBXT-positive cells (49%) within these matrix deposition hubs (Figures 4D, 4H, 4I, S4B, S4E, and S4F), supporting the interpretation that TGF-β1 promotes maturation of pre-specified notochord-biased cells, rather than introducing heterogeneity, in line with the work by Tang et al. (2018) and Tong et al. (2024) (Tang et al., 2018; Tong et al., 2024). However, TBXT quantification was performed on tissue sections, which may not fully reflect the entire pellet. Due to similar technical constraints, the matrix deposition hub was not captured for TBXT staining in NS9 pellets (Figure S4B).

These findings demonstrate that 3D culture enhances cell maturation and matrix deposition in CRISPRa-differentiated iPSCs. Notably, SOX-trio showed superior mRNA expression of matrix genes compared to NS9 and NTS9. Extended culture periods will likely be required for more complete differentiation, as seen in iPSC-based cartilage differentiation protocols (Adkar et al., 2019; Rodríguez Ruiz et al., 2021).

DISCUSSION

This work presents a stepwise differentiation pipeline using the well-established CRISPRa-SAM system (Konermann et al., 2015) to identify a minimal TF combination to direct iPSCs toward the NC lineage. This pipeline includes: (1) testing multiple gRNAs for each gene to optimize dCas9 targeting, (2) combining gRNAs in a pTri-gRNA construct to generate specific gRNA combinations, (3) using fluorescent markers to track transfection and differentiation, and (4) employing SORT-seq to characterize distinct cell populations. Guided by a developmental biology rationale, six gene in eight combinations were targeted. Single-cell transcriptome analysis revealed that *SOX5*, *SOX6*, and *SOX9* as the minimal combination, produced the largest notochord population. Furthermore, using the ACAN-2A-mScarlet iPSC reporter line to track aggrecan-producing cells, we show in 3D pellet culture that iNLCs form a matrix-enriched region composed of aggrecan-producing cells, enriched in TBXT-positive cells, demonstrating functional maturation through matrix deposition.

We first successfully reproduced iMEPCs through WNT activation (70% TBXT+/FOXA2+), consistent with earlier reports (Tong et al., 2024; Warin et al., 2024). While CRISPRa of NOTO worked in iMEPCs, these *NOTO*-activated iMEPCs did not exhibit significant upregulation of key NC markers, such as *SHH*, or functional markers, *ACAN* and *COL2A1*. In contrast, iMEPCs subjected to the *NOTO+TBXT+FOXA2* combination showed notable expression of these NC and functional markers, indicating the synergistic role of *NOTO*, *TBXT*, and *FOXA2* in promoting notochord formation (Yamanaka et al., 2007). Warin et al. (2024) reported high SOX9 expression in iNLCs, supporting its key regulatory role in notochord formation as shown in developmental studies (Yamanaka et al., 2007; Warin et al., 2024). The inclusion of *SOX9* in the *NOTO+TBXT+FOXA2* combination resulted in higher expression of *SHH*, *ACAN*, and *COL2A1* compared to *NOTO+TBXT+FOXA2* (Smits and Lefebvre 2003). Interestingly, the activation of the *SOX* genes stimulated comparable expression of key NC and functional markers to *NOTO+SOX9* and *NOTO+TBXT+SOX9* but achieved significantly higher expression compared to *NOTO+TBXT+FOXA2*. These results underscore the pivotal role of *SOX* genes in the NC lineage commitment ultimately leading to healthy matrix-producing cells.

It should be noted that notochord formation requires both tightly regulated and inherently stochastic transcriptional events within an interacting TF network (Nakamichi

and Asahara 2020). The variability in gene expression observed in this study likely is confounded by differences in cell state or stage of differentiation, as well as variations in CRISPRa transfection efficiency and locus-specific activation efficiency. In addition, simultaneous activation of multiple TFs may produce non-linear interactions, such as partial compensation or feedback modulation, resulting in differing expression amplitudes across targets without altering overall lineage bias (Takeuchi et al., 2011). These factors may contribute to persistent cellular heterogeneity. Nevertheless, the differentiation pipeline established here provides a framework to approximate the coordinated transcriptional modulation that occurs during early development. In combination with FACS sorting of CRISPRa transfected cells, this pipeline supports the optimization of iNLC differentiation.

Complementary single-cell transcriptome analysis revealed that SOX-trio activation generated the largest notochord cluster (13.5%–21.9% of the differentiated iPSCs), accounting for over 90% of the cells in the notochord cluster identified by single-cell analysis. These findings highlight the strong effect of coordinated *SOX* genes activation in promoting iNLC differentiation. It's worth noting that *SOX* genes are also essential for chondrogenesis, driving matrix gene expression such as *ACAN* (Han and Lefebvre 2008). However, the transcriptome profile and TF regulatory networks underlying notochord versus cartilage developmental trajectories are distinct. In this study, SOX-trio activation occurs in a $TBXT^{+}$/$FOXA2^{+}$ mesoendoderm context under BMP inhibition, which biases their activity toward a notochordal rather than chondrogenic trajectory. Consistently, single-cell sequencing of day-7 differentiated cells showed that *TBXT* was undetectable, while *FOXA2* expression persisted in a distinct notochord cluster expressing previously reported notochord-associated markers, including *SHH*, *FOXA1*, *FOXA2*, *FOXJ1*, *FN1*, *ALCAM*, *KRT8*, and *KRT18*, amid the mesodermal progenitor clusters, in line with previous studies (Tong et al., 2024; Warin et al., 2024). Furthermore, the presence of *FOXA2* in the absence of *RUNX2*, *COL10*, and *MMP13* argues against the induction of hypertrophic chondrocytes, and supports *FOXA2* expression being confined to a juvenile context especially the notochord (Ionescu et al., 2012; Bell et al., 2022).

Regardless of the CRISPRa-mediated transactivation, the lineage heterogeneity prevailed in all three minimal TF combinations. Single-cell sequencing revealed four distinct mesoderm clusters, mesoderm progenitors-1, -2, -3, and neuromesoderm, a phenomenon commonly observed in other iPSC differentiation strategies (Tang et al., 2018; Colombier et al., 2020; Zhang et al., 2020; Warin et al., 2024). Rather than reflecting failed transactivation, this likely represents the inherent variability of lineage commitment, where cells respond differently depending on their state and microenvironment. To control transfection efficiency, the *mNeonGreen* sequence in the pTri-gRNA plasmid enabled identification of CRISPRa-transfected cells and their subsequent FACS purification one day post-transfection. This ensured that only transfected cells were analyzed, thereby excluding heterogeneity caused by variable plasmid uptake. In our previous study using the same delivery approach (Tong et al., 2024), all CRISPR-activated cells were contained within the population marked by a co-delivered GFP reporter plasmid, demonstrating strong overlap between reporter positivity and functional CRISPR activation. Therefore, it is reasonable to interpret the mNeonGreen-positive population as a reliable indicator of cells co-transfected with both CRISPRa plasmids. Notably, the CRISPRa effect was transient, with mNeonGreen tracking showing reduced signal after approximately five days. This reflects the short-lived nature of plasmid-based expression in iPSCs. Nevertheless, these transient transcriptional activations were sufficient to drive secondary shifts in lineage-associated genes, suggesting that brief but coordinated TF activity can bias lineage specification. Attempts to prolong activation through multiple rounds of mRNA transfection, as reported by Colombier et al. (2020), did not demonstrate additional benefit (Colombier et al., 2020). Therefore, a single CRISPRa transfection was adopted in this proof-of-concept study preventing cellular stress of multiple transfections within the established differentiation framework.

While CRISPR off-target binding events can theoretically occur, they are largely defined by sequence-dependent hot spots and would be expected to occur similarly across different gRNA combinations. Thus, the heterogeneity observed here more likely reflects differentiation plasticity rather than CRISPR off-target activity. Furthermore, SORT-seq allowed us to correlate the fluorescent signal with transcriptome-defined cell clusters, enabling a more precise characterization of cell identities (Muraro et al., 2016). The mNeonGreen-positive cells were detected within the heterogeneous populations across all three groups, with the lowest accumulating in the neuromesoderm cluster. A plausible explanation is that these bipotent progenitor cells further differentiate into posterior neural and paraxial mesoderm lineages, utilizing TF networks that overlap with those driving NC lineage commitment (Sambasivan and Steventon 2021). Notably, Warin et al. (2024) reported that inhibition of the TGF-β pathway with a selective inhibitor can reduce lineage heterogeneity and enhance iNLC differentiation (Warin et al., 2024). In the present study, we chose to focus on the TF network driving iNLC differentiation rather than applying pathway inhibition, allowing us to assess heterogeneity arising solely from intrinsic lineage commitment and

TF-mediated regulation. The robust expression of notochord markers observed with the SOX-trio combination may be attributed to a paracrine effect exerted by the mixed mesoderm progenitor cells during iNLC differentiation. These mixed mesoderm progenitor clusters likely mimic the early embryonic or developing NP environment, which could play a supportive role in iNLC differentiation. This indicates that the mixed population may be inevitable for notochord cluster generation. While the behavior and regulation of these neuromesoderm-like cells and their derivatives within *in vitro* differentiation models are underexplored and beyond the scope of this work, tracking mNeonGreen cells offers valuable insights. Rather than eliminating heterogeneity, these findings highlight the need for strategies that harness mixed populations to refine lineage commitment.

Using the ACAN-2A-mScarlet iPSC reporter line, we evaluated the minimal TF combinations—NS9, NS9, and SOX-trio—and found that CRISPRa-transfected cells exhibited enhanced maturation in 3D culture compared with monolayer condition. This was evident from the increased mScarlet-positive population, significant upregulation of functional and reporter genes, and substantial matrix deposition at the protein level. It's noteworthy that these matrix-producing cells may be associated with iNLCs, as indicated by the substantial overlap between ACAN-producing cells and TBXT-positive cells in the matrix-rich region. In the present study, iPSCs were differentiated sequentially into iMEPCs and directed toward the mesoderm lineage with the emergence of a distinct notochord population through CRISPRa-mediated modulation evidenced by single-cell sequencing. Based on this foundation, TGF-β1 treatment was introduced during the 3D culture phase. Therefore, it's reasonable to infer that TGF-β1 primarily facilitated the maturation of iNLCs instead of increasing heterogeneity. Interestingly, the matrix-producing cells form a spatially distinct hub adjacent to the main pellet rather than a uniform spherical structure, the latter commonly seen in iPS-chondrogenesis (Adkar et al., 2019; van Hoolwerff et al., 2021; Tong et al., 2024). It is tempting to hypothesize that this spatial organization may reflect underlying cell-cell or cell-matrix interactions during early notochord development. These interactions drive clustering of notochord-biased cells from the primitive node migrating cranially to form the notochordal process, whereafter the notochordal process extends forward, subsequently transforming into the notochordal plate, and ultimately folding into the definitive notochord (Corallo et al., 2015; Bach et al., 2021).

Consistent with single-cell sequencing results, SOX-trio activation proved to be the most efficient in guiding iNLC differentiation. Larger scale TF screening using this pipeline is warranted to refine the differentiation protocol. In addition, whilst 3D pellet cultures showed increased NC marker expression such as *NOGGIN*, *COL2A1*, and *ACAN*, along with distinct GAG-rich matrix production, they lacked the native disc's complex biomechanical and biochemical cues. Culturing differentiated cells in a disc-like environment may enhance iNLC maturation. This caveat could also explain the absence of vacuolated NCs in both monolayer and 3D pellet differentiation; the morphogenesis and maintenance of vacuolation happens at the late stages of notochord formation and requires complex spatiotemporal signal regulation in embryonic development (Wang et al., 2017). Given this, a deeper investigation of the transcriptome profile of the native NCs and its comparison with iNLCs is warranted. Comparative analysis has been reported previously with limited NCs number (Warin et al., 2024), underscoring the need for expanded studies.

Once the ideal minimal set of TFs is defined, precise regulation through a CRISPR-dCas9 activation system could offer a promising strategy to improve iNLC differentiation. Within this context, CRISPRa DNA transfection has slower kinetics, as the transcription and translation steps delay Cas9 and gRNA activity, increasing the risk of off-target effects (Lin et al., 2022). In contrast, CRISPRa delivery via mRNA or protein transfection offers immediate and transient CRISPR activity (Kim et al., 2014), and are safer alternatives in future studies, as they avoid the risk of integration into the host genome.

In summary, we report the first successful application of the CRISPRa-SAM system, combining single-cell sorting and analysis, to establish a robust platform for refining differentiation protocols for generating iNLCs. This CRISPRa system enables multiplexed gene modulation, revealing the *SOX5+SOX6+SOX9* combination (SOX-trio) as essential for promoting NC lineage commitment. Using the ACAN-2A-mScarlet iPSC reporter line, CRISPRa-transfected cells were further matured in 3D culture and in the presence of TGF-β1 into aggrecan-producing cells, as evidenced by detectable mScarlet expression. The dual use of the mNeonGreen reporter in the CRISPRa-SAM system and mScarlet reporter iPSC line enabled precise tracking of both transfection and differentiation processes, with future potential for cell purification. CRISPR activation of iPSCs remains technically challenging even beyond the field of iNLC differentiation. Overall, this integrated platform represents a significant technical advancement toward optimizing CRISPR-based differentiation strategies, addressing the heterogeneity that remains a challenge in iPSC-derived notochordal differentiation.

RESOURCE AVAILABILITY

Lead contact

Further information and requests for resources and reagents should be directed to and will be fulfilled by Deepani W. Poramba-Liyanage (deepsliyanage@gmail.com).

Materials availability

The pTri-gRNA construct was generated by and is available from Dr. Peng Shang. No other new unique reagents were generated in this study.

Data and code availability

The Raw RNA sequencing data have been submitted to NCBI Gene Expression Omnibus (GEO) and can be accessed under the accession number GSE283877. This paper does not report original code.

ACKNOWLEDGMENTS

This work was funded by European Union's Horizon 2020 research and innovation program (iPSpine; grant agreement number 825925); Friends of Veterinary Medicine fund, housed at Stichting Utrechts Universiteitsfonds (grant number 2610577) and China Scholarship Council (CSC). We thank Megan Woering and Georgios Dimitrios Chatzoglou for their contribution of making the pTri-gRNA constructs with SOX-trio combination. We thank Amir Kamali for his expert evaluation of the histology staining and providing valuable pathological insights. We thank the FACS facility of the Hubrecht Institute for their support and expertise, and single cell discovery, Utrecht, the Netherlands for performing the single-cell RNA sequencing.

AUTHOR CONTRIBUTIONS

X.T., M.V., N.G., P.S., M.A.T., and D.W.P.-L. conceived the project. X.T., M.V., D.V., and D.W.P.-L. performed experiments and analyzed the data. P.S. provided the CRISPRa plasmids. F.M.R. performed single-cell RNA sequencing analysis. X.T., M.A.T., and D.W.P.-L. wrote the original manuscript. All authors read the manuscript, provided feedback, and approved the final manuscript.

DECLARATION OF INTERESTS

The authors declare no competing interest.

STAR★METHODS

Detailed methods are provided in the online version of this paper and include the following:

- KEY RESOURCES TABLE
- EXPERIMENTAL MODEL AND STUDY PARTICIPANT DETAILS
  - iPSC culture
- METHOD DETAILS
  - Generation of pTri-gRNA constructs
  - gRNA selection
  - iNLC differentiation
  - Reverse transcription quantitative PCR
  - Immunofluorescence staining and histology staining
  - Single-cell RNA sequencing
  - Single-cell RNA seq data analysis
- QUANTIFICATION AND STATISTICAL ANALYSIS
  - Statistical analysis

SUPPLEMENTAL INFORMATION

Supplemental information can be found online at https://doi.org/10.1016/j.stemcr.2026.103060.

Received: September 3, 2025
Revised: July 29, 2026
Accepted: July 30, 2026
Published: August 27, 2026

REFERENCES

Adkar, S.S., Wu, C.L., Willard, V.P., Dicks, A., Ettyreddy, A., Steward, N., Bhutani, N., Gersbach, C.A., and Guilak, F. (2019). Step-Wise Chondrogenesis of Human Induced Pluripotent Stem Cells and Purification Via a Reporter Allele Generated by CRISPR-Cas9 Genome Editing. Stem Cell. *37*, 65–76. https://doi.org/10.1002/stem.2931.

Alini, M., Eisenstein, S.M., Ito, K., Little, C., Kettler, A.A., Masuda, K., Melrose, J., Ralphs, J., Stokes, I., and Wilke, H.J. (2008). Are animal models useful for studying human disc disorders/degeneration? Eur. Spine J. *17*, 2–19. https://doi.org/10.1007/s00586-007-0414-y.

Bach, F.C., Poramba-Liyanage, D.W., Riemers, F.M., Guicheux, J., Camus, A., Iatridis, J.C., Chan, D., Ito, K., Le Maitre, C.L., and Tryfonidou, M.A. (2021). Notochordal Cell-Based Treatment Strategies and Their Potential in Intervertebral Disc Regeneration. Front. Cell Dev. Biol. *9*, 780749. https://doi.org/10.3389/fcell.2021.780749.

Barrionuevo, F., Taketo, M.M., Scherer, G., and Kispert, A. (2006). Sox9 is required for notochord maintenance in mice. Dev. Biol. *295*, 128–140. https://doi.org/10.1016/j.ydbio.2006.03.014.

Bell, N., Bhagat, S., Muruganandan, S., Kim, R., Ho, K., Pierce, R., Kozhemyakina, E., Lassar, A.B., Gamer, L., Rosen, V., and Ionescu, A.M. (2022). Overexpression of transcription factor FoxA2 in the developing skeleton causes an enlargement of the cartilage hypertrophic zone, but it does not trigger ectopic differentiation in immature chondrocytes. Bone *160*, 116418. https://doi.org/10.1016/j.bone.2022.116418.

Burridge, P.W., Thompson, S., Millrod, M.A., Weinberg, S., Yuan, X., Peters, A., Mahairaki, V., Koliatsos, V.E., Tung, L., and Zambidis, E.T. (2011). A universal system for highly efficient cardiac differentiation of human induced pluripotent stem cells that eliminates interline variability. PLoS One *6*, e18293. https://doi.org/10.1371/journal.pone.0018293.

Butler, A., Hoffman, P., Smibert, P., Papalexi, E., and Satija, R. (2018). Integrating single-cell transcriptomic data across different conditions, technologies, and species. Nat. Biotechnol. *36*, 411–420. https://doi.org/10.1038/nbt.4096.

Cheung, K.M.C., Samartzis, D., Karppinen, J., and Luk, K.D.K. (2012). Are patterns of lumbar disc degeneration associated with low back pain?: new insights based on skipped level disc pathology. Spine *37*, E430–E438. https://doi.org/10.1097/BRS.0b013e3182304dfc.

Choi, K.-S., and Harfe, B.D. (2011). Hedgehog signaling is required for formation of the notochord sheath and patterning of nuclei

pulposi within the intervertebral discs. Proc. Natl. Acad. Sci. USA *108*, 9484–9489. https://doi.org/10.1073/pnas.1007566108.

Colombier, P., Halgand, B., Chédeville, C., Chariau, C., François-Campion, V., Kilens, S., Vedrenne, N., Clouet, J., David, L., Guicheux, J., and Camus, A. (2020). NOTO Transcription Factor Directs Human Induced Pluripotent Stem Cell-Derived Mesendoderm Progenitors to a Notochordal Fate. Cells *9*, 509. https://doi.org/10.3390/cells9020509.

Corallo, D., Trapani, V., and Bonaldo, P. (2015). The notochord: structure and functions. Cell. Mol. Life Sci. *72*, 2989–3008. https://doi.org/10.1007/s00018-015-1897-z.

Diwan, A.D., and Melrose, J. (2023). Intervertebral disc degeneration and how it leads to low back pain. JOR Spine *6*, e1231. https://doi.org/10.1002/jsp2.1231.

Fausett, S.R., Brunet, L.J., and Klingensmith, J. (2014). BMP antagonism by Noggin is required in presumptive notochord cells for mammalian foregut morphogenesis. Dev. Biol. *391*, 111–124. https://doi.org/10.1016/j.ydbio.2014.02.008.

Hafemeister, C., and Satija, R. (2019). Normalization and variance stabilization of single-cell RNA-seq data using regularized negative binomial regression. Genome Biol. *20*, 296. https://doi.org/10.1186/s13059-019-1874-1.

Han, Y., and Lefebvre, V. (2008). L-Sox5 and Sox6 drive expression of the aggrecan gene in cartilage by securing binding of Sox9 to a far-upstream enhancer. Mol. Cell Biol. *28*, 4999–5013. https://doi.org/10.1128/MCB.00695-08.

Hunter, C.J., Matyas, J.R., and Duncan, N.A. (2004). Cytomorphology of notochordal and chondrocytic cells from the nucleus pulposus: a species comparison. J. Anat. *205*, 357–362. https://doi.org/10.1111/j.0021-8782.2004.00352.x.

Ionescu, A., Kozhemyakina, E., Nicolae, C., Kaestner, K.H., Olsen, B.R., and Lassar, A.B. (2012). FoxA family members are crucial regulators of the hypertrophic chondrocyte differentiation program. Dev. Cell *22*, 927–939. https://doi.org/10.1016/j.devcel.2012.03.011.

Kim, S., Kim, D., Cho, S.W., Kim, J., and Kim, J.-S. (2014). Highly efficient RNA-guided genome editing in human cells via delivery of purified Cas9 ribonucleoproteins. Genome Res. *24*, 1012–1019. https://doi.org/10.1101/gr.171322.113.

Konermann, S., Brigham, M.D., Trevino, A.E., Joung, J., Abudayyeh, O.O., Barcena, C., Hsu, P.D., Habib, N., Gootenberg, J.S., Nishimasu, H., et al. (2015). Genome-scale transcriptional activation by an engineered CRISPR-Cas9 complex. Nature *517*, 583–588. https://doi.org/10.1038/nature14136.

Lefebvre, V., and Dvir-Ginzberg, M. (2017). SOX9 and the many facets of its regulation in the chondrocyte lineage. Connect. Tissue Res. *58*, 2–14. https://doi.org/10.1080/03008207.2016.1183667.

Lin, Y., Wagner, E., and Lächelt, U. (2022). Non-viral delivery of the CRISPR/Cas system: DNA versus RNA versus RNP. Biomater. Sci. *10*, 1166–1192. https://doi.org/10.1039/d1bm01658j.

Luoma, K., Riihimäki, H., Luukkonen, R., Raininko, R., Viikari-Juntura, E., and Lamminen, A. (2000). Low back pain in relation to lumbar disc degeneration. Spine *25*, 487–492. https://doi.org/10.1097/00007632-200002150-00016.

Maeder, M.L., Linder, S.J., Cascio, V.M., Fu, Y., Ho, Q.H., and Joung, J.K. (2013). CRISPR RNA–guided activation of endogenous human genes. Nat. Methods *10*, 977–979. https://doi.org/10.1038/nmeth.2598.

McInnes, L., Healy, J., and Melville, J. (2018). Umap: Uniform manifold approximation and projection for dimension reduction. arXiv. https://doi.org/10.48550/arXiv.1802.03426.

Muhl, L., Genové, G., Leptidis, S., Liu, J., He, L., Mocci, G., Sun, Y., Gustafsson, S., Buyandelger, B., Chivukula, I.V., et al. (2020). Single-cell analysis uncovers fibroblast heterogeneity and criteria for fibroblast and mural cell identification and discrimination. Nat. Commun. *11*, 3953. https://doi.org/10.1038/s41467-020-17740-1.

Mullally, G., Van Aelst, K., Naqvi, M.M., Diffin, F.M., Karvelis, T., Gasiunas, G., Siksnys, V., and Szczelkun, M.D. (2020). 5′ modifications to CRISPR–Cas9 gRNA can change the dynamics and size of R-loops and inhibit DNA cleavage. Nucleic Acids Res. *48*, 6811–6823. https://doi.org/10.1093/nar/gkaa477.

Muraro, M.J., Dharmadhikari, G., Grün, D., Groen, N., Dielen, T., Jansen, E., van Gurp, L., Engelse, M.A., Carlotti, F., de Koning, E.J.P., and van Oudenaarden, A. (2016). A Single-Cell Transcriptome Atlas of the Human Pancreas. Cell Syst. *3*, 385–394.e3. https://doi.org/10.1016/j.cels.2016.09.002.

Nakamichi, R., and Asahara, H. (2020). The transcription factors regulating intervertebral disc development. JOR Spine *3*, e1081. https://doi.org/10.1002/jsp2.1081.

Nakami, W.N., J., Nguhiu-Mwangi, A.N.e., Kipyegon, M., Ogugo, C., Muteti, Kemp, S., 2022. Comparative efficiency for in vitro transfection of goat undifferentiated spermatogonia using lipofectamine reagents and electroporation. Stem Cell. Clon Adv. Appl. 15, 11–20. https://doi.org/10.2147/sccaa.s356588.

Nelms, B.L., and Labosky, P.A. (2010). Transcriptional Control of Neural Crest Development. Morgan & Claypool Life Sciences. https://doi.org/10.4199/c00010ed1v01y201003deb001.

Ng, A.H.M., Khoshakhlagh, P., Rojo Arias, J.E., Pasquini, G., Wang, K., Swiersy, A., Shipman, S.L., Appleton, E., Kiaee, K., Kohman, R.E., et al. (2021). A comprehensive library of human transcription factors for cell fate engineering. Nat. Biotechnol. *39*, 510–519. https://doi.org/10.1038/s41587-020-0742-6.

Revilla, A., González, C., Iriondo, A., Fernández, B., Prieto, C., Marín, C., and Liste, I. (2016). Current advances in the generation of human iPS cells: implications in cell-based regenerative medicine. J. Tissue Eng. Regen. Med. *10*, 893–907. https://doi.org/10.1002/term.2021.

Rodríguez Ruiz, A., Dicks, A., Tuerlings, M., Schepers, K., van Pel, M., Nelissen, R.G.H.H., Freund, C., Mummery, C.L., Orlova, V., Guilak, F., et al. (2021). Cartilage from human-induced pluripotent stem cells: comparison with neo-cartilage from chondrocytes and bone marrow mesenchymal stromal cells. Cell Tissue Res. *386*, 309–320. https://doi.org/10.1007/s00441-021-03498-5.

Sambasivan, R., and Steventon, B. (2020). Neuromesodermal progenitors: a basis for robust axial patterning in development and evolution. Front. Cell Dev. Biol. *8*, 607516. https://doi.org/10.3389/fcell.2020.607516.

Sanson, K.R., Hanna, R.E., Hegde, M., Donovan, K.F., Strand, C., Sullender, M.E., Vaimberg, E.W., Goodale, A., Root, D.E., Piccioni, F., and Doench, J.G. (2018). Optimized libraries for CRISPR-Cas9 genetic screens with multiple modalities. Nat. Commun. *9*, 5416. https://doi.org/10.1038/s41467-018-07901-8.

Semprich, C.I., Davidson, L., Amorim Torres, A., Patel, H., Briscoe, J., Metzis, V., and Storey, K.G. (2022). ERK1/2 signalling dynamics promote neural differentiation by regulating chromatin accessibility and the polycomb repressive complex. PLoS Biol. *20*, e3000221. https://doi.org/10.1371/journal.pbio.3000221.

Sive, J.I., Baird, P., Jeziorsk, M., Watkins, A., Hoyland, J.A., and Freemont, A.J. (2002). Expression of chondrocyte markers by cells of normal and degenerate intervertebral discs. Mol. Pathol. *55*, 91–97. https://doi.org/10.1136/mp.55.2.91.

Smits, P., and Lefebvre, V. (2003). Sox5 and Sox6 Are Required for Notochord Extracellular Matrix Sheath Formation, Notochord Cell Survival and Development of the Nucleus Pulposus of Intervertebral Discs. Development *130*, 1135–1148. https://doi.org/10.1242/dev.00331.

Takahashi, K., and Yamanaka, S. (2006). Induction of pluripotent stem cells from mouse embryonic and adult fibroblast cultures by defined factors. cell *126*, 663–676.

Takeuchi, J.K., Lou, X., Alexander, J.M., Sugizaki, H., Delgado-Olguín, P., Holloway, A.K., Mori, A.D., Wylie, J.N., Munson, C., Zhu, Y., et al. (2011). Chromatin remodelling complex dosage modulates transcription factor function in heart development. Nat. Commun. *2*, 187. https://doi.org/10.1038/ncomms1187.

Tang, R., Jing, L., Willard, V.P., Wu, C.L., Guilak, F., Chen, J., and Setton, L.A. (2018). Differentiation of human induced pluripotent stem cells into nucleus pulposus-like cells. Stem Cell Res. Ther. *9*, 61. https://doi.org/10.1186/s13287-018-0797-1.

Tong, X., Poramba-Liyanage, D.W., van Hoolwerff, M., Riemers, F.M., Montilla-Rojo, J., Warin, J., Salvatori, D., Camus, A., Meulenbelt, I., Ramos, Y.F.M., et al. (2024). Isolation and tracing of matrix-producing notochordal and chondrocyte cells using ACAN-2A-mScarlet reporter human iPSC lines. Sci. Adv. *10*, eadp3170. https://doi.org/10.1126/sciadv.adp3170.

van Hoolwerff, M., Rodríguez Ruiz, A., Bouma, M., Suchiman, H.E.D., Koning, R.I., Jost, C.R., Mulder, A.A., Freund, C., Guilak, F., Ramos, Y.F.M., and Meulenbelt, I. (2021). High-impact FN1 mutation decreases chondrogenic potential and affects cartilage deposition via decreased binding to collagen type II. Sci. Adv. *7*, eabg8583. https://doi.org/10.1126/sciadv.abg8583.

Wang, F., Gao, Z.-X., Cai, F., Sinkemani, A., Xie, Z.-Y., Shi, R., Wei, J.-N., and Wu, X.-T. (2017). Formation, function, and exhaustion of notochordal cytoplasmic vacuoles within intervertebral disc: current understanding and speculation. Oncotarget *8*, 57800–57812. https://doi.org/10.18632/oncotarget.18101.

Warin, J., Vedrenne, N., Tam, V., Zhu, M., Yin, D., Lin, X., Guidoux-D'halluin, B., Humeau, A., Roseiro, L., Paillat, L., et al. (2024). In vitro and in vivo models define a molecular signature reference for human embryonic notochordal cells. iScience *27*, 109018. https://doi.org/10.1016/j.isci.2024.109018.

Wu, A., March, L., Zheng, X., Huang, J., Wang, X., Zhao, J., Blyth, F.M., Smith, E., Buchbinder, R., and Hoy, D. (2020). Global low back pain prevalence and years lived with disability from 1990 to 2017: estimates from the Global Burden of Disease Study 2017. Ann. Transl. Med. *8*, 299. https://doi.org/10.21037/atm.2020.02.175.

Yamanaka, Y., Tamplin, O.J., Beckers, A., Gossler, A., and Rossant, J. (2007). Live imaging and genetic analysis of mouse notochord formation reveals regional morphogenetic mechanisms. Dev. Cell *13*, 884–896. https://doi.org/10.1016/j.devcel.2007.10.016.

Zhang, Y., Zhang, Z., Chen, P., Ma, C.Y., Li, C., Au, T.Y.K., Tam, V., Peng, Y., Wu, R., Cheung, K.M.C., et al. (2020). Directed Differentiation of Notochord-like and Nucleus Pulposus-like Cells Using Human Pluripotent Stem Cells. Cell Rep. *30*, 2791–2806.e5. https://doi.org/10.1016/j.celrep.2020.01.100.

Zhao, C.-Q., Wang, L.-M., Jiang, L.-S., and Dai, L.-Y. (2007). The cell biology of intervertebral disc aging and degeneration. Ageing Res. Rev. *6*, 247–261. https://doi.org/10.1016/j.arr.2007.08.001.

STAR★METHODS

KEY RESOURCES TABLE

REAGENT or RESOURCE	SOURCE	IDENTIFIER
**Antibodies**		
Goat polyclonal anti-Brachyury (TBXT)	Novus Biologicals	Cat# AF2085; RRID: AB_2200235
Rabbit monoclonal anti-FOXA2	Cell Signaling	Cat# 8186S; RRID: AB_10891055
Mouse monoclonal anti-panKRT	Abcam	Cat# ab41825; RRID: AB_736438
Rabbit polyclonal anti-Aggrecan	Santa Cruz	Cat# sc-25674; RRID: AB_2288908
Mouse monoclonal anti-mScarlet	Synaptic Systems	Cat# 409011; RRID: AB_2800533
Alexa Fluor 488 goat anti-mouse IgG (H+L)	Invitrogen	Cat# A11029; RRID: AB_2534088
Alexa Fluor 488 goat anti-rabbit IgG (H+L)	Invitrogen	Cat# A32731; RRID: AB_2633280
Alexa Fluor 568 goat anti-rabbit IgG (H+L)	Invitrogen	Cat# A11036; RRID: AB_10563566
Alexa Fluor 568 goat anti-mouse IgG (H+L)	Invitrogen	Cat# A11004; RRID: AB_2534072
Alexa Fluor 568 donkey anti-goat IgG (H+L)	Abcam	Cat# ab175704; RRID: AB_2725786
**Bacterial and virus strains**		
5-alpha competent *E. coli* (High efficiency)	NEB	Cat# C2987
**Chemicals, peptides, and recombinant proteins**		
SuRE/Cut buffer M	Roche	Cat# 11417983001
BsaI-HF	NEB	Cat# R3733
BbsI	NEB	Cat# R0539
Esp3I	NEB	Cat# R0734
T4 DNA Ligase	Thermo Scientific	Cat# EL0011
Matrigel Matrix	Corning	Cat# 354277
mTeSR™1	STEMCELL Technologies	Cat# 85850
PBS	Gibco	Cat# 10010-015
UltraPure™ 0.5M EDTA	Invitrogen	Cat# 15575020
TrypLE™ Express Enzyme	Gibco	Cat# 12604-013
Y-27632 dihydrochloride	Tocris Bioscience	Cat# 1254
Lipofectamine STEM	Invitrogen	Cat# STEM00008
Laminin 521	Corning	Cat# 354223
CHIR99021	Axon	Cat# 1386
DMEM/F-12	Gibco	Cat# 31331028
MEM Non-Essential Amino Acids Solution	Gibco	Cat# 11140050
GlutaMAX™ Supplement	Gibco	Cat# 35050061
2-Mercaptoethanol	Gibco	Cat# 31350010

(*Continued on next page*)

Continued

REAGENT or RESOURCE	SOURCE	IDENTIFIER
N2 Supplement	Gibco	Cat# 17502048
B27 Supplement	Gibco	Cat# 17504044
Bovine Serum Albumin	Sigma-Aldrich	Cat# A1595
Human Noggin	Miltenyi Biotec	Cat# 130-103-456
DMEM, high glucose, GlutaMAX^{TM} Supplement	Gibco	Cat# 31966
ITS+ Premix Universal Culture Supplement	Corning	Cat# 354352
L-Proline	Sigma-Aldrich	Cat# P5607
L-Ascorbic acid 2-phosphate sesquimagnesium salt hydrate	Sigma-Aldrich	Cat# A8960
Recombinant Human TGF-beta 1 Protein	R&D Systems	Cat# 240-B-010
4% paraformaldehyde	Klinipath	Cat# 4078.9010
Triton^{TM} X-100	Sigma-Aldrich	Cat# T8787
DAPI solution	Thermo Scientific	Cat# 62248
FluorSave Reagent	EMD Millipore	Cat# 345789
Mayer's hemalum solution	Sigma-Aldrich	Cat# 1.09249
Eosin Y	Sigma-Aldrich	Cat# 1.15935
Alcian Blue 8GX	Sigma-Aldrich	Cat# 05500
Fast Green FCF	Sigma-Aldrich	Cat# F7252
**Critical commercial assays**		
QIAquick Gel Extraction Kit	Qiagen	Cat# 28704
QIAprep Spin Miniprep Kit	Qiagen	Cat# 27106
PureLink^{TM} HiPure Plasmid Maxiprep Kit	Invitrogen	Cat# K210007
RNeasy Mini Kit	Qiagen	Cat# 74106
RNeasy Micro Kit	Qiagen	Cat# 74004
iScript cDNA Synthesis Kit	Bio-rad	Cat# 1708891
iQ^{TM} SYBR® Green Supermix	Bio-rad	Cat# 1708886
**Deposited data**		
Single-cell RNA sequencing	This study	GEO: GSE283877
**Experimental models: Cell lines**		
Human episomel iPSC line; TMOi001-A	Gibco	Cat# A18945; RRID: CVCL_RM92
ACAN-2A-mScarlet reporter iPSC line; TMOi001-A-14	Tong et al. (2024)	N/A
**Oligonucleotides**		
gRNA library: See Table S1	Konermann et al. (2015), Sanson et al. (2018)	N/A
Primers used in this study: See Table S6	This study	N/A

(*Continued on next page*)

Continued

REAGENT or RESOURCE	SOURCE	IDENTIFIER
**Recombinant DNA**		
PB-SAM-Dest vector	Addgene	102563; RRID: Addgene_102563
pTri-gRNA	This study	N/A
**Software and algorithms**		
SnapGene	Snapgene	RRID: SCR_015052
Fiji imageJ	Open-source	RRID: SCR_002285
Adobe Illustrator	Adobe	RRID: SCR_010279
Adobe Photoshop	Adebe	RRID: SCR_014199
Illumina DRAGEN BCL Convert	Illumina, Inc	Version 4.1.23
Cutadapt	Open-source	Version 4.7; RRID: SCR_011841
Ribodetector	Open-source	Version 0.3.1
STARsolo	Open-source	Version 2.7.10b; RRID: SCR_021542
RStudio	RStudio Team	Version 1.2.5019; RRID: SCR_000432
R	R core Team	version 3.6.1; RRID: SCR_001905
GraphPad Prism 9	GraphPad	RRID: SCR_002798

EXPERIMENTAL MODEL AND STUDY PARTICIPANT DETAILS

iPSC culture

Human Episomal iPSC line (TMOi001-A) (Burridge et al., 2011) and ACAN-2A-mScarlet reporter iPSCs (TMOi001-A-14, generated by the Department of Anatomy and Embryology, Leiden University Medical Center) were cultured on 6-well cell culture plates coated with Matrigel. An in-frame *mScarlet* reporter gene was introduced downstream of the endogenous *ACAN* CDS, and between the CDS of *ACAN* and *mScarlet*, a *2A* self-cleaving peptide sequence was placed to allow the formation of separated proteins of these two genes. The TMOi001-A-14 iPSC line has been characterized for the maintenance of pluripotency using qPCR analysis of pluripotency markers and by assessing its capacity for multilineage differentiation (Tong et al., 2024). The genomic integrity has been confirmed by karyotyping, and the reporter function has been validated using CRISPR-mediated transactivation of the *ACAN* gene (Tong et al., 2024). mTeSR1 was used for iPSC maintenance culture and the medium was changed daily. iPSCs were passaged every 4 to 5 days at approximately 80% confluency. Cells were washed twice with PBS and detached by incubating with a 0.5 mM EDTA solution (0.5 M EDTA diluted in PBS) for 5 minutes at room temperature.

METHOD DETAILS

Generation of pTri-gRNA constructs

The CRISPRa-SAM system is composed of three components: 1). dCas9-VP64, a fusion protein of the nuclease-deficient Cas9 (dCas9) and the VP64 activation domain, which consists of four tandem VP16 (Herpes Simplex Viral Protein 16) domain; 2). MS2-P65-HSF1, a chimeric protein which enhances transcription independently from VP64, contains a hair-pin RNA binding protein MS2, a P65 transactivation subunit of NF-κB and a heat shock transcription factor 1 (HSF1); 3). a modified guide RNA (gRNA) containing multiple MS2 RNA stem-loops, which binds to Cas9 to form RNP as well as recruit the MS2-P65-HSF1 chimeric protein. For guide delivery, the pTri-gRNA construct allows for simultaneous expression of 3 separate guides via three distinct promoters (H1 promoter expressing guide 1; 7SK promoter expressing guide 2, and U6 promoter expressing guide 3).

The mNeonGreen reporter sequence was integrated into the pTri-gRNA vector (pTri-gRNA kindly provided by Dr. Peng Shang) to check the transfection efficiency. The commonly used promoters for gRNA transcription have similar initiation requirements such as gRNAs expressed from U6 promoter and need to start with a G or A for high expression (Mullally et al., 2020). Therefore, an extra "G" was added to each guide inserting site in front of the 5′ side of each guide sequence. 3 to 9 single guides were designed based on the published genome-wide libraries for CRISPRa (Konermann et al., 2015; Sanson et al., 2018) (Table S1). The gRNA sequences were produced by Eurogentec, and the single-stranded oligos were resuspended in SuRE/Cut^{TM} buffer M into 100 μM concentration. The forward and reverse oligos of each guide were mixed together and kept at 100°C for 2 minutes to anneal them together. Then, the annealed double-stranded fragments were gradually cooled to 4°C at 0.1°C/second for temporary storage. Restriction enzymes were used to digest the three different guide-inserting sites in the pTri-gRNA vector following the commercial restriction digestion protocols, using BsaI for the H1 promoter site, BbsI for the 7SK promoter site and Esp3I for the U6 promoter site. Agarose gel electrophoresis and gel extraction (with Qiagen gel extraction kit) were performed to purify the digested vectors. The annealed gRNA sequences were mixed with the digested linear vector at a molar ratio of 3:1 (insert: vector). T4 DNA Ligase was used to ligate the annealed guide oligos and pTri-gRNA linear vectors with 1 hour incubation at room temperature. Transformation of DH5α competent cells with the ligated vectors was performed to select and replicate the successfully ligated vectors with the different inserting gRNA sequences. The vector DNA was isolated by miniprep kit and tested by Sanger sequencing to confirm the correct insertion of the guide sequences. After the sequencing, further purification and replication of the integrated vectors was performed by maxiprep kit following the manufacturer's instructions and the vectors' DNA was diluted into 1 μg/ml as the final concentration for storage.

gRNA selection

To identify potent gRNAs enabling the CRISPRa-SAM-mediated gene activation, the gRNA integrating pTri-gRNA vector was co-transfected with the PB-SAM-Dest vector into human TMOi001-A iPSCs. To select the best performed gRNA of each gene efficiently, a primary test of the gRNAs of each gene was performed in iPSCs with electroporation. 80% confluent iPSCs were dissociated by TrypLE and 1 million iPSCs were electroporated with 4 μg well-mixed CRISPRa plasmids (PB-SAM-Dest + pTri-gRNA, the molar ratio is 1:1) using NEPA21 Electroporator (150V poring pulse for 5ms, 20V transfer pulse for 50 ms). The electroporated iPSCs were seeded back on Matrigel coated 12-well plates at 160,000 cells/cm^2 in mTeSR1 medium supplemented with ROCK inhibitor, and cells were collected for RT-qPCR 48 hours after electroporation. Due to the lower cell survival and higher cytotoxicity of electroporation compared to the lipofectamine approach (Nakami et al., 2022), a secondary test of the best gRNAs was performed in iPSCs with lipofectamine transfection. The iPSCs were seeded on Matrigel-coated 12-well plates at 50,000 cells/cm^2 in mTeSR1 medium supplemented with ROCK inhibitor on day 0. DNA transfection was carried out within 24 hours using Lipofectamine STEM following the commercial protocol. Transfected cells were collected on day 3 for RT-qPCR analysis. The final selected gRNAs were used in the following differentiation process.

iNLC differentiation

The human TMOi001-A iPSC line and TMOi001-A-14 reporter iPSC line were differentiated into the notochordal lineage as described by Warin et al. (2024) (Warin et al., 2024). Briefly, the human iPSCs were seeded on Laminin 521 coated plates and chamber slides at 20,000 cells/cm^2 in mTeSR1 medium supplemented with ROCK inhibitor on day -2. After two days of recovery, iPSCs were cultured in N2B27 medium (described in Table S5) supplemented with 3 μM CHIR99021 from day 0 to day 2 to activate the WNT pathway and drive iPSCs to iMEPCs.

The iMEPCs were dissociated with TrypLE and transfected in single-cell suspension with CRISPRa plasmids using Lipofectamine STEM at a DNA:Lipofectamine volume ratio of 1:5 (1.5 μg total DNA per 6-well, 156 ng/cm^2), based on the manufacturer's recommendations and our previous study with CRISPRa transfection (Tong et al., 2024). Cells were subsequently seeded on new Laminin521-coated plates at 160,000 cells/cm^2. iMEPCs that were not transfected with CRISPRa plasmids were used as nontransfection controls. The CRISPRa transfected iMEPCs and non-transfected iMEPCs were maintained in N2B27 medium supplemented with 3 μM CHIR99021 complemented with 100 ng/mL recombinant human Noggin for 5 days allowing the emergence of embryonic iNLCs and cells were collected daily for RT-qPCR. A 3D culture follow up step was performed to mature the differentiated iNLCs in the presence of TGF-β1, with day 7 monolayer cells dissociated and pelleted (1,200 rpm, 5 mins) in round bottom nonadherent 96-well plates (Costar, #7007) at 200,000 cells per pellet and well. Pellets were maintained in disc medium (hgDMEM + Glutamax with 1% ITS Premix, 1% MEM Non-essential amino acids (NEAA), 40 μg/ml L-Proline, 0.1 mM ascorbic acid 2-phosphate (Asap)) together with 10 ng/mL TGF-β1 for 21 days. The differentiation process was performed at 37°C with 5% CO_2 and 20% O_2. Medium was refreshed daily.

Reverse transcription quantitative PCR

To examine the mRNA expression of pluripotency markers, mesendoderm markers, and notochordal markers, the total RNA from monolayer cells was extracted using RNeasy mini kit and RNA from pellets was extracted using RNeasy micro kit. The cDNA was then synthesized using iScript cDNA synthesis kit for quantitative reverse transcription PCR detection. Relative quantification was calculated using the efficiency-corrected $\Delta\Delta C_T$ method. For each target gene, the C_T value was normalised to the mean C_T value of the reference genes (*GAPDH* and *YWHAZ*) using the following formula: $\Delta C_T = C_{T\ target} - C_{T\ mean\ ref}$. As a calibrator, the ΔC_T value of initial iPSCs was used for statistical analysis. Thereafter, the $E^{-\Delta\Delta CT}$ value for the test and calibrator sample was calculated to determine relative expression. In this formula, E indicates the amplification efficiency (between 95% and 105%) of the target gene. The primer sequences are listed in Table S6.

Immunofluorescence staining and histology staining

The monolayer differentiated cells cultured in chamber slides were fixed in 4% paraformaldehyde (PFA) for 10 minutes. The fixed cells were incubated for 1 hour with PBS containing 0.1% Triton X-100 and 3% BSA to permeabilize and block unspecific binding sites. After washing with PBS twice, these cells were incubated with primary antibodies: TBXT (0.5 μg/mL, 1:400), FOXA2 (50 kDa, 1:400), panKRT (1 μg/mL, 1:100), Aggrecan (2 μg/mL, 1:100) and mScarlet (2 μg/mL, 1:500) at 4°C overnight. After washing with PBS three times, the slides were incubated with the secondary antibodies: Goat anti-Mouse IgG (H+L), Alexa Fluor 488 (1:1000), Goat anti-Rabbit IgG (H+L), Alexa Fluor 488 (1:1000), Goat anti-Rabbit IgG (H+L), Alexa Fluor 568 (1:1000), Donkey anti-Goat IgG (H+L), Alexa Fluor 568 (1:1000) and Goat anti-Mouse IgG (H+L), Alexa Fluor 568 (1:1000) in dark for one hour at room temperature. After washing with PBS for three times, cells were incubated with DAPI for 10 minutes at room temperature. Finally, the slides were mounted by fluorsave reagent and imaged using a confocal microscope (Leica TCS SP8 X).

The pellet samples were fixed and stained in 4% PFA containing 0.1% Eosin overnight at room temperature. After fixation, the pellets were embedded in 3% agarose. The solidified agarose droplets, with pellet samples, were paraffin-embedded using the tissue processor (Leica ASP300S). The paraffin-embedded sections were cut at a thickness of 5 μm and collected on positively charged microscopy slides (VWR, #631-1185). The pellet morphology and matrix deposition were assessed by histological staining using H&E and Alcian Blue staining following previously established protocols. TBXT, KRT8/18/19 (panKRT), Aggrecan and mScarlet markers were determined by immunofluorescence staining described above.

Single-cell RNA sequencing

SORT-seq, a method based on fluorescence-activated cell sorting (FACS) followed by high-throughput scRNA-seq was performed in day 7 differentiated iPSCs. To efficiently capture single-cell transcriptomes, a single-cell sequencing platform based on the CEL-Seq2 protocol, based on cytometry-based cell sorting and robotics liquid handling was performed by Single Cell Discoveries (The Netherlands) as described before (Muraro et al., 2016). Single-cell library preparations, Illumina sequencing, and read alignment to the human genome (CanFam 3.1) were performed by Single Cell Discoveries as described in Muraro et al. (2016) (Muraro et al., 2016). Briefly, cells were washed with PBS three times and concentrated to 700-1,200 cells per μL. The cDNA libraries were sequenced on an Illumina Novaseq™ X Plus. Demultiplexing was performed using Illumina's DRAGEN BCL Convert software (version 4.1.23). The resulting reads were preprocessed as follows: Illumina sequencing adapters were removed and reads containing homopolymers were trimmed using cutadapt (version 4.7). Ribosomal reads were discarded using ribodetector (version 0.3.1). A custom reference genome was built using the GRCh38 assembly and annotation from Ensembl release 98, incorporating the sequences and annotations for two reporter genes, *mNeonGreen* and *mScarlet* (<insert transgene's source>).

Technical replicates were generated for each TF combination. For the SOX-trio condition, two biological replicates were performed: the first biological replicate consisted of a single dataset (SOX-trio-1), while the second biological replicate consisted of two technical replicates (SOX-trio-2 and SOX-trio-3), which were pooled for downstream analysis. Cells were sorted into plates across multiple experimental days. To minimise batched-associated variation, all plates were processed in parallel through cDNA synthesis, library preparation, and sequencing.

Single-cell RNA seq data analysis

An assessment of potential batch effects was performed by visualizing UMAP embeddings annotated with condition and plate metadata. Clustering did not segregate by plate of origin; hence no computational batch correction or dataset integration was applied. The preprocessed reads were aligned to the custom reference genome using STARsolo (version 2.7.10b), and count tables were generated. Reads mapping to multiple genomic locations were discarded and

then further processed in Rstudio (version 1.2.5019; R Studio Team, 2020) and R (version 3.6.1; R Core Team, 2019) using the Seurat package (Butler et al., 2018). Cells expressing <1,000 or >10,000 genes, <30,000 Unique Molecular Identifier counts, and <10 percent mitochondrial genes were filtered out. Next, data were normalised and scaled with the SCTransform method,(Hafemeister and Satija 2019) and principle component analysis was used for dimension reduction. Based on the elbow plot, the first 15 principle components were used for the clustering analysis. Clusters were visualized with Uniform Manifold Approximation and Projection (UMAP) (McInnes et al., 2018). Differentially expressed genes between clusters were obtained using a non-parametric Wilcoxon rank sum test at a significance level of $p < 0.05$. Data was normalised and scaled with the SCTransform method (Hafemeister and Satija 2019).

Data Availability: The raw RNA sequencing data has been submitted to NCBI Gene Expression Omnibus (GEO) and can be accessed under the accession number GSE283877.

QUANTIFICATION AND STATISTICAL ANALYSIS

Statistical analysis

Statistical analysis was performed using GraphPad Prism 9. Data passed the normal distribution test. Parametric t-test was performed comparing two groups in the gRNAs test. One-way ANOVA with Benjamini-Hochberg correction was performed in the multiple groups' comparison in iNLC differentiation. Each quantification was derived from a minimum of three independent samples. A p-value of <0.05 was considered statistically significant. All average values described in the text and figures are mean ± SD (standard deviation).

Stem Cell Reports, Volume *21*

Supplemental Information

CRISPR activation reveals *SOX5/6/9* as key transcriptional regulators directing iPSC-derived cells toward a notochordal lineage

Xiaole Tong, Marieke Visscher, Frank M. Riemers, Danielle Versluis, Niels Geijsen, Peng Shang, Marianna A. Tryfonidou, and Deepani W. Poramba-Liyanage

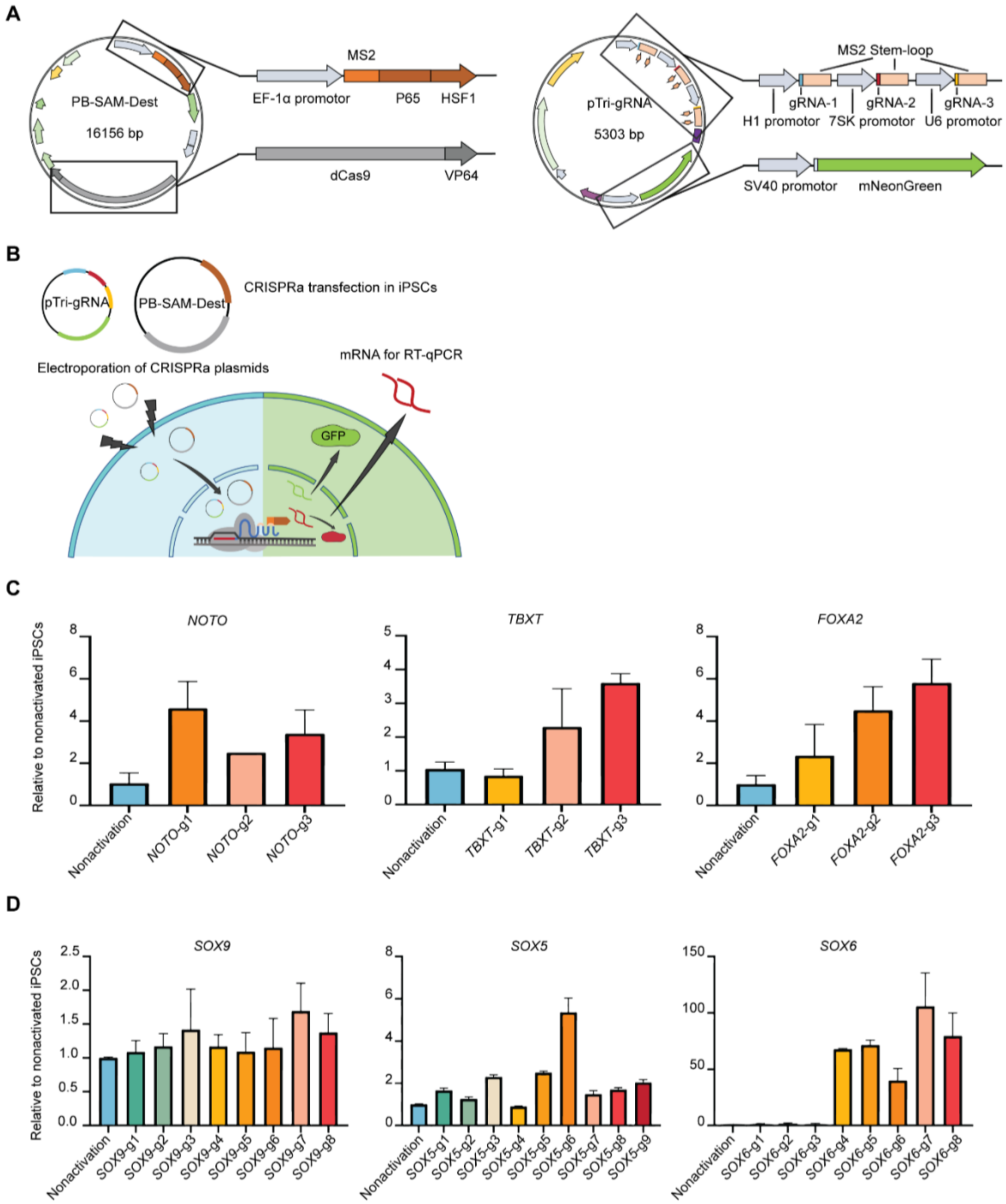

Figure S1. CRISPRa-mediated induction of target genes in iPSCs, related to Figure 1

(A) Schematic of constructs used to introduce the CRISPRa-SAM system and guide RNAs into cells, together with *mNeonGreen*. The CRISPR construct delivers the dCas9, and components of the CRISPRa-SAM system, the VP64 proteins, P65, HSF1 and the MS2 coat protein. The pTri-gRNA construct that allows for the expression of 3 separate guides via its promoter was used for guide delivery; with the H1 promoter expressing guide 1, the 7SK promoter expressing guide 2, and the U6 promoter expressing guide 3. An SV40 promoter that drives the expression of *mNeonGreen* is introduced with the pTri-gRNA construct.

(B) gRNA selection pipeline. Human iPSCs were introduced with the PB-SAM-Dest construct together with the pTri-gRNA construct targeting different genomic locations by electroporation, and cells were collected for RT-qPCR.

(C and D) Preliminary gRNA screening for *NOTO*, *TBXT*, *FOXA2*, *SOX9*, *SOX5* and *SOX6*. Data are presented as mean ± SD, n=2 biological replicates (independent transfections). This screen was used to rank gRNAs, whereafter selected guides were subsequently validated in triplicate with statistical analysis (see Figure 1F).

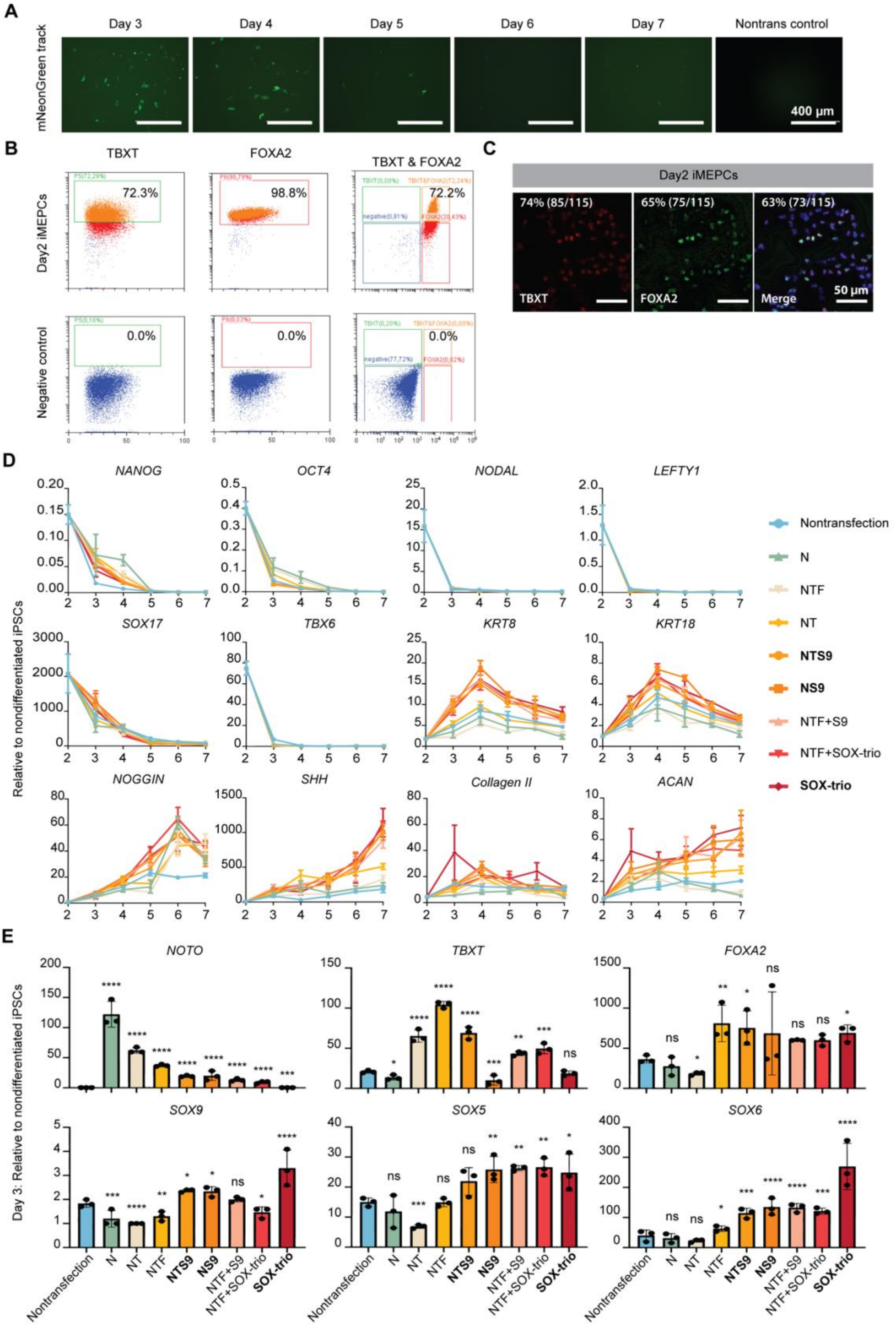

A
Day 3
Day 4
Day 5
Day 6
Day 7
Nontrans control
mNeonGreen track
400 μm
B
TBXT
FOXA2
TBXT & FOXA2
Day2 iMEPCs
Negative control
72.3%
98.8%
72.2%
0.0%
0.0%
0.0%
C
Day2 iMEPCs
74% (85/115)
65% (75/115)
63% (73/115)
TBXT
FOXA2
Merge
50 μm
D
NANOG
OCT4
NODAL
LEFTY1
SOX17
TBX6
KRT8
KRT18
NOGGIN
SHH
Collagen II
ACAN
Relative to nondifferentiated iPSCs
Nontransfection
N
NTF
NT
NTS9
NS9
NTF+S9
NTF+SOX-trio
SOX-trio
E
NOTO
TBXT
FOXA2
SOX9
SOX5
SOX6
Day 3: Relative to nondifferentiated iPSCs
Nontransfection
N
NT
NTF
NTS9
NS9
NTF+S9
NTF+SOX-trio
SOX-trio

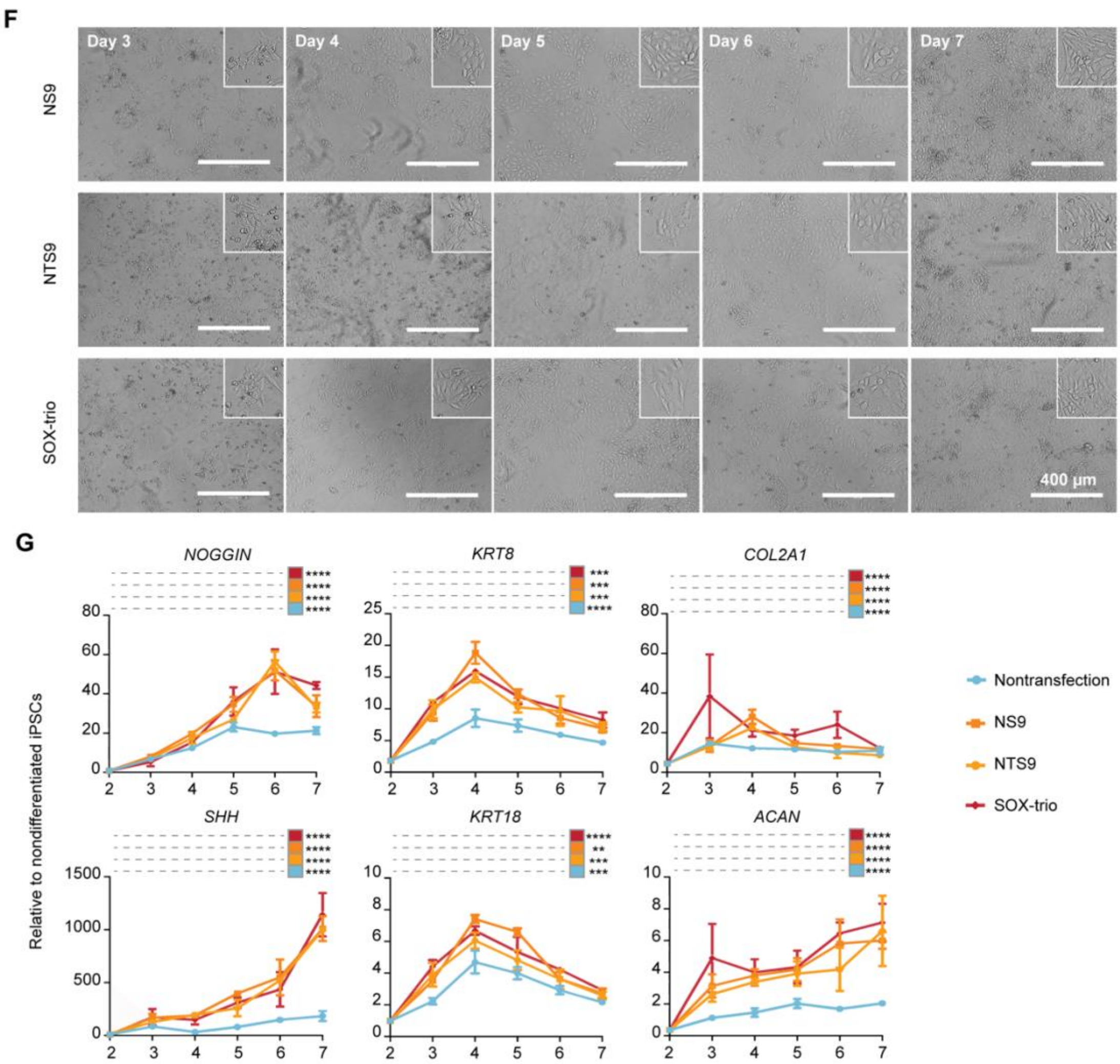

Figure S2. CRISPRa-mediated induction of target genes to promote NC lineage commitment, related to Figure 2

(A) mNeonGreen expression coupled to the pTri-gRNA plasmid as a readout for transfection efficiency. mNeonGreen expression remained detectable up to 5 days after transfection into iMEPCs.

(B) FACS measurement of day 2 iMEPCs showing induction of TBXT single positive; FOXA2 single positive and TBXT and FOXA2 double positive cell populations after WNT pathway activation.

(C) IF staining of day 2 iMEPCs for TBXT and FOXA2 positive populations.

(D) Time course expressions of pluripotency markers (*NANOG*, *OCT4*), WNT pathway markers (*NODAL* and *LEFTY1*), endoderm (*SOX17*), mesoderm marker (*TBX6*) and notochordal markers (*KRT8, KRT18, NOGGIN, SHH, COL2A1 and ACAN*) with comparable gene expression profiles in all groups over the culture period. Data presented as mean ± SD, n=3 biological replicates generated independently.

(E) mRNA levels of CRISPRa targeted genes in day 3 iMEPCs (24 hours after CRISPRa transfection) show successful gene activation. Significance indicated at the day 3 CRISPRa transfected cells compared with day 3 non-transfected (nontrans) cells.

(F) Cell morphology changes overtime after CRISPRa transfection in NTS9, NS9 and SOX-trio groups.

(G) Time course expressions of all key notochordal genes in Nontransfection, NTS9, NS9 and SOX-trio groups. Significance indicated at the day 7 differentiated iPSCs compared with day 2 iMEPCs. Data presented as mean ± SD, *P<0.05, **P<0.01, ***P<0.001, ***P<0.0001, ns: no significance, n=3 biological replicates generated independently.

Scalebars: 400 µm in A and F; 50 µm in C.

N: *NOTO* guides; NT: *NOTO*-g1+*TBXT*-g3; NTF: *NOTO*-g1+*TBXT*-g3+*FOXA2*-g3; NS9: *NOTO*-g1+*SOX9*-g7; NTS9: *NOTO*-g1+*TBXT*-g3+*SOX9*-g7; NTF+S9: *NOTO*-g1+*TBXT*-g3+*FOXA2*-g3+*SOX9*-g7; SOX-trio: *SOX9*-g7+*SOX5*-g6+*SOX6*-g7; NTF+SOX-trio: *NOTO*-g1+*TBXT*-g3+*FOXA2*-g3+ *SOX9*-g7+*SOX5*-g6+*SOX6*-g7

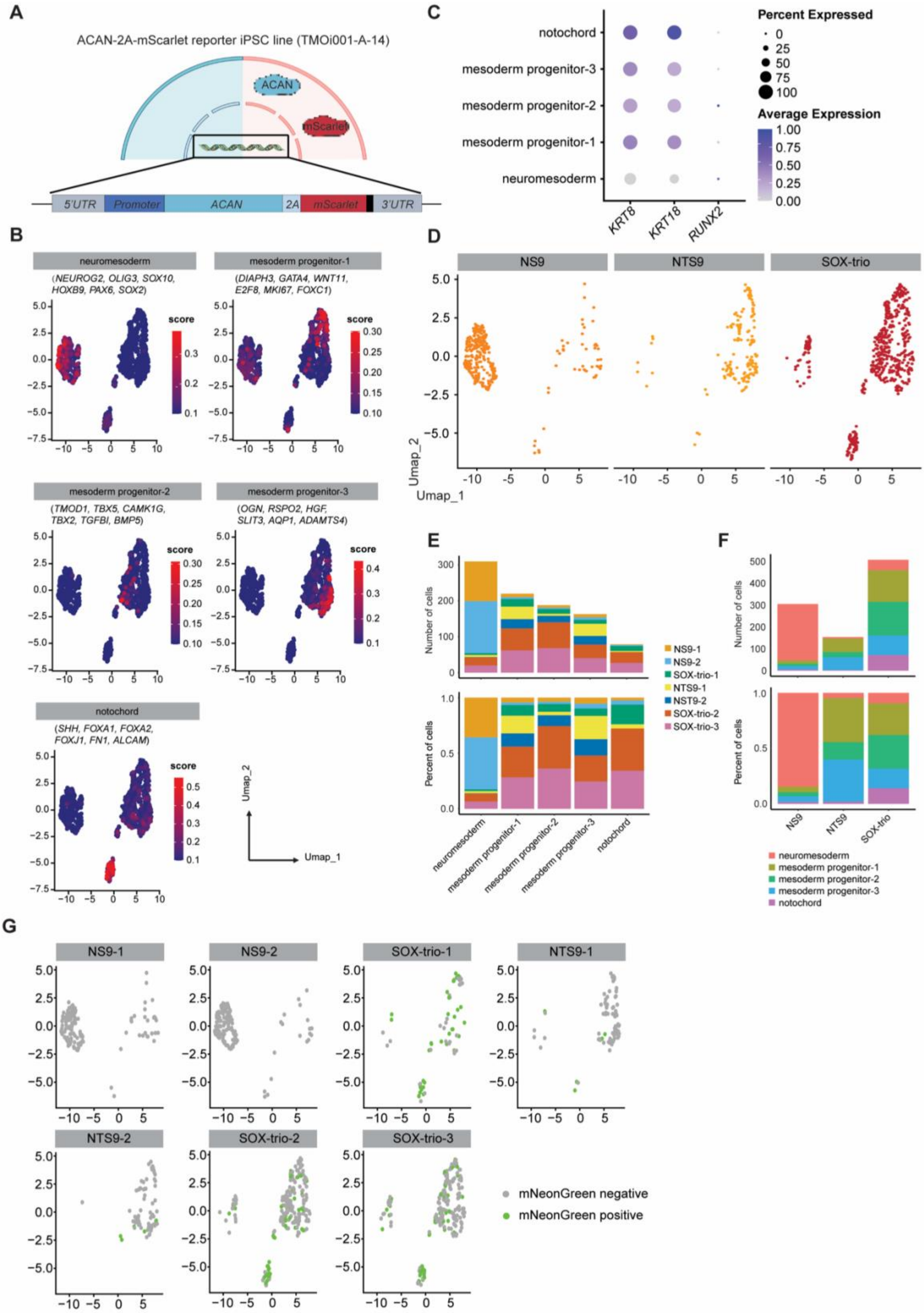
A
ACAN-2A-mScarlet reporter iPSC line (TMOi001-A-14)
ACAN
mScarlet
5'UTR
Promoter
ACAN
2A
mScarlet
3'UTR
B
neuromesoderm
(NEUROG2, OLIG3, SOX10, HOXB9, PAX6, SOX2)
mesoderm progenitor-1
(DIAPH3, GATA4, WNT11, E2F8, MKI67, FOXC1)
mesoderm progenitor-2
(TMOD1, TBX5, CAMK1G, TBX2, TGFBI, BMP5)
mesoderm progenitor-3
(OGN, RSPO2, HGF, SLIT3, AQP1, ADAMTS4)
notochord
(SHH, FOXA1, FOXA2, FOXJ1, FN1, ALCAM)
score
Umap_2
Umap_1
C
notochord
mesoderm progenitor-3
mesoderm progenitor-2
mesoderm progenitor-1
neuromesoderm
KRT8
KRT18
RUNX2
Percent Expressed
0
25
50
75
100
Average Expression
1.00
0.75
0.50
0.25
0.00
D
NS9
NTS9
SOX-trio
Umap_2
Umap_1
E
Number of cells
Percent of cells
NS9-1
NS9-2
SOX-trio-1
NTS9-1
NST9-2
SOX-trio-2
SOX-trio-3
neuromesoderm
mesoderm progenitor-1
mesoderm progenitor-2
mesoderm progenitor-3
notochord
F
Number of cells
Percent of cells
NS9
NTS9
SOX-trio
neuromesoderm
mesoderm progenitor-1
mesoderm progenitor-2
mesoderm progenitor-3
notochord
G
NS9-1
NS9-2
SOX-trio-1
NTS9-1
NTS9-2
SOX-trio-2
SOX-trio-3
mNeonGreen negative
mNeonGreen positive

Figure S3. Single-cell RNA sequencing highlights the key contribution of the SOX-trio to NC lineage commitment, related to Figure 3

(A) The schematic of the ACAN-2A-mScarlet reporter iPSC line (TMOi001-A-14).

(B) Identification of key clusters based on the average combined marker score using UCell.

(C) Scaled dot plots of mean normalised expression for *KRT8*, *KRT18* and *RUNX2* genes per cluster.

(D) UMAP-based plots indicating the contribution of each gRNA combination activated by the CRISPRa-SAM system with the visualized five clusters.

(E and F) Contribution of gRNA combinations and sequencing run to each cluster.

(G) UMAP plots displaying scaled *mNeonGreen* intensity (0–100) after quality filtering. For NS9 and NTS9, fewer *mNeonGreen*-positive cells passed filtering, consistent with lower retained viability. Technical replicates were generated for each TF combination. For the SOX-trio condition, two biological replicates were performed: SOX-trio-1 represents the first biological replicate, while SOX-trio-2 and SOX-trio-3 are technical replicates from the second biological replicate and were pooled for downstream analyses in Figure 3.

Technical replicates were generated for each transcription-factor combination. For the SOX-trio condition, two biological replicates were performed: the first biological replicate consisted of a single dataset (SOX-trio-1), while the second biological replicate consisted of two technical replicates (SOX-trio-2 and SOX-trio-3), which were pooled for downstream analysis.

NS9: *NOTO*-g1+*SOX9*-g7; NTS9: *NOTO*-g1+*TBXT*-g3+*SOX9*-g7; SOX-trio: *SOX9*-g7+*SOX5*-g6+*SOX6*-g7

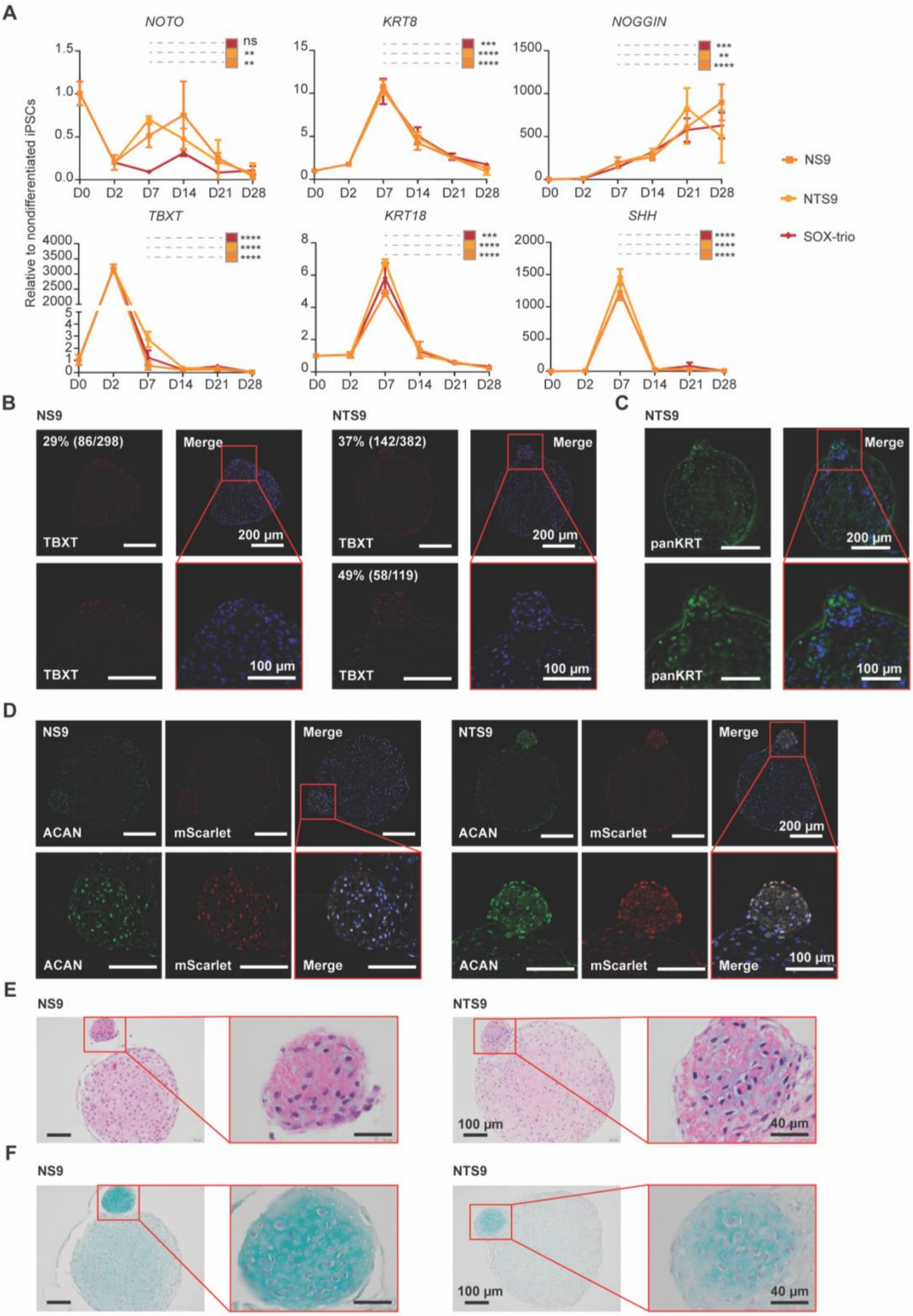

Figure S4. Matrix deposition by iPSCs promoted to the NC lineage through CRISPRa during 3D pellet culture, related to Figure 4

(A) Gene expression of *NOTO*, *TBXT*, *KRT8, KRT18, NOGGIN* and *SHH* during the monolayer and 3D pellet culture period. Significance indicated at day 28 CRISPRa transfected pellets compared with day 7 monolayer cells. Data presented as mean ± SD, **P<0.01, ***P<0.001, ****P<0.0001, ns: no significance, n=3 biological replicates (generated independently).

(B) Representative immunofluorescence staining of TBXT of day 28 NS9 and NTS9 pellets.
(C) Representative immunofluorescence staining of panKRT of day 28 NTS9 pellet.
(D) Representative immunofluorescence staining of ACAN and mScarlet of day 28 NS9 and NTS9 pellets.
(E and F) Representative H&E and Alcian blue staining images of the day 28 NS and NTS9 pellets showing a matrix-rich core.
Scalebars: 200 µm in top images and 100 µm in bottom images in B, C and D; 100 µm in left images and 40 µm in right images in E and F.
NS9: *NOTO*-g1+*SOX9*-g7; NTS9: *NOTO*-g1+*TBXT*-g3+*SOX9*-g7; SOX-trio: *SOX9*-g7+*SOX5*-g6+*SOX6*-g7

Table S1: List of CRISPRa gRNA sequences, related to Figure 1 and S1

Genes	Guide sequence	Library
NOTO-g1	GTGAGCGCCCGCTCTGTGCG	Sanson, K. R. et al. (2018)
NOTO-g2	CCGCTCTGTGCGAGGCAAGG	Sanson, K. R. et al. (2018)
NOTO-g3	CACCTTGCCTCGCACAGAGC	Sanson, K. R. et al. (2018)
TBXT-g1	GAGTGTGCCAGGCGTGTGCG	Sanson, K. R. et al. (2018)
TBXT-g2	CGGAAGGTGGGAGAAGGTCG	Sanson, K. R. et al. (2018)
TBXT-g3	GTCATTGCTTTCACGGAAGG	Sanson, K. R. et al. (2018)
FOXA2-g1	CTCTCGCGCTCCCTCTCCCT	Sanson, K. R. et al. (2018)
FOXA2-g2	AGAGGGAGCGCGAGAGAGGG	Sanson, K. R. et al. (2018)
FOXA2-g3	GGGAGGAGGGGACGGTGCTT	Sanson, K. R. et al. (2018)
SOX9-g1	ATCACAGCACAGCAGTCCCA	Sanson, K. R. et al. (2018)
SOX9-g2	CACTGGGCTGGGTGACGAGG	Sanson, K. R. et al. (2018)
SOX9-g3	CAGCACAGCAGTCCCAGGGT	Sanson, K. R. et al. (2018)
SOX9-g4	TGGCACTGGGCTGGGTGACG	Sanson, K. R. et al. (2018)
SOX9-g5	ATCAGCTGCCTGCCAACCCT	Sanson, K. R. et al. (2018)
SOX9-g6	CCTCCTCCCTCCCCAAAATC	Sanson, K. R. et al. (2018)
SOX9-g7	AATCAGCTGCCTGCCAACCC	Konermann, S. et al. (2015)
SOX9-g8	GATCGCAGCCAAAGGGCGGA	Konermann, S. et al. (2015)
SOX5-g1	TAGCCCCCAGTTAATCACTT	Konermann, S. et al. (2015)
SOX5-g2	TCAGTTGATTAGATTTTCTG	Konermann, S. et al. (2015)
SOX5-g3	TCTGGAAATGGAGCAGACAA	Konermann, S. et al. (2015)
SOX5-g4	CCTCGTCCTTGAGAGCAACA	Sanson, K. R. et al. (2018)
SOX5-g5	TCCCTTTGCTGGTCATGAGG	Sanson, K. R. et al. (2018)
SOX5-g6	TCGTCCTTGAGAGCAACACG	Sanson, K. R. et al. (2018)
SOX5-g7	AAATCCCTTTGCTGGTCATG	Sanson, K. R. et al. (2018)
SOX5-g8	CCGTGTTGCTCTCAAGGACG	Sanson, K. R. et al. (2018)
SOX5-g9	GCCGCCTCATGACCAGCAAA	Sanson, K. R. et al. (2018)
SOX6-g1	CCTCAGATCACCTTCAACTT	Konermann, S. et al. (2015)
SOX6-g2	GCCTCTTGCACCAAAGTTGA	Konermann, S. et al. (2015)
SOX6-g3	TGTCCTTTATAAAGGCAAAA	Konermann, S. et al. (2015)
SOX6-g4	TCTTGCCTGTGTTGTCTGGG	Sanson, K. R. et al. (2018)
SOX6-g5	GCTCCCCTCCCAGACAACAC	Sanson, K. R. et al. (2018)
SOX6-g6	TTGCCTGTGTTGTCTGGGAG	Sanson, K. R. et al. (2018)
SOX6-g7	CAAGATGAGACAAGAGGCGA	Sanson, K. R. et al. (2018)
SOX6-g8	CTTGCCTGTGTTGTCTGGGA	Sanson, K. R. et al. (2018)

Table S2: Set up of the iNLC differentiation in this study, related to Figure 2-4 and S2-S4

Part of Study	Cell State	gRNAs	FACS	TGF-β1	Note
Stage 1 (Broad screen) 7 days in monolayer culture	iPSCs → iMEPCs → CRISPRa guides sets	N, NT, NTF, NTS9, NS9, NTF+S9, NTF+SOX-trio, SOX-trio	No sorting	No	Detect initial lineage bias and select best-performing guides
Stage 2 (High-resolution lineage mapping) 7 days in monolayer culture	Same differentiation as in Stage 1 but with CRISPRa-transfected cells purification	NS9, NTS9, SOX-trio	FACS sorted for CRISPRa activation reporter	No	Define cluster identity and co-expression structure
Stage 3 (Maturation) 7 days in monolayer and 21 days in 3D pellet	Day-7 cultures aggregated into pellets	NS9, NTS9, SOX-trio	No sorting	With TGF-β1 in 3D pellet	Assess matrix-producing phenotype and maturation trajectory

Table S3: Cell distribution in clusters by plate after filtering, related to Figure 3 and S3

Cluster	NS9-1	NS9-2	**NS9 total**	NTS9-1	NTS9-2	**NTS9 total**	SOX-trio-1*	SOX-trio-2	SOX-trio-3	**SOX-trio total**
neuromesoderm	111	145	**256**	6	1	**7**	5	22	20	**47**
ratio	79.9%	88.4%	**84.5%**	7.1%	1.4%	**4.6%**	7.8%	9.7%	9.2%	**9.3%**
mesoderm progenitor-1	10	5	**15**	35	26	**61**	21	61	62	**144**
ratio	7.2%	3.0%	**5.0%**	41.7%	37.7%	**39.9%**	32.8%	27.0%	28.6%	**28.4%**
mesoderm progenitor -2	7	4	**11**	6	18	**24**	13	72	68	**153**
ratio	5.0%	2.4%	**3.6%**	7.1%	26.1%	**15.7%**	20.3%	31.9%	31.3%	**30.2%**
mesoderm progenitor -3	9	7	**16**	34	24	**58**	11	38	40	**89**
ratio	6.5%	4.3%	**5.3%**	40.5%	34.8%	**37.9%**	17.2%	16.8%	18.4%	**17.6%**
notochord	2	3	**5**	3	0	**3**	14	33	27	**74**
ratio	1.4%	1.8%	**1.7%**	3.6%	0.0%	**2.0%**	21.9%	14.6%	12.4%	**14.6%**
total	139	164	**303**	84	69	**153**	64	226	217	**507**

Technical replicates were processed for each transcription factor combination sorted on separate plates for single cell sequencing. A biological replicate was conducted for SOX-trio. Note that for technical reasons data was not available from the first technical replicate of SOX-trio-1, and SOX-trio-2 and SOX-trio-3 are second technical replicate.

Table S4: *mNeonGreen*-positive cells distribution after filtering, related to Figure 3 and S3

Conditions	neuro mesoderm	mesoderm progenitor -1	mesoderm progenitor -2	mesoderm progenitor -3	notochord
Total cell number	310	220	188	163	82
mNeonGreen+	12	30	40	16	44
ratio	3.9%	13.6%	21.3%	9.8%	53.7%
mNeonGreen-	298	190	148	147	38
ratio	96.1%	86.4%	78.7%	90.2%	46.3%

Table S5: Medium preparation, related to Figure 2-3 and S2-S3

N2B27 MEDIUM				
**COMPONENT**	**Supplier**	**Identifier**	**Stock concentration**	**Final concentration**
DMEM/F-12	Gibco	31331028	-	-
NEAA	Gibco	11140050	100X	1X
GLUTAMAX	Gibco	35050061	100X	1X
BETA-MERCAPTOETHANOL	Gibco	31350010	50 mM	0.001
N2 SUPPLEMENT	Gibco	17502048	100X	0.5X
B27 SUPPLEMENT	Gibco	17504044	50X	0.5X
BOVINE ALBUMIN	Sigma	A1595	0.1	0.00005

Table S6: List of qPCR primers, related to Figure 1, 2, 4, S1, S2 and S4

Genes	Forward sequence	Reverse sequence	Tm (°C)
NANOG	CCAGAGAATGAAATCTAAGAGGTG	CAGGTTGAATTGTTCCAGGT	60
OCT4	GAGAACCGAGTGAGAGGCAACC	CATAGTCGCTGCTTGATCGCTTG	60
NODAL	TGGAAGGCTGGGAAACTC	CTCCTCTTCAGTTCTGGTGG	57
LEFTY1	CATCGAGGGACTTGACTTGTG	AGAGCATTTGTCCATCAGCAG	63.5
SOX17	TCTGCCTCCTCCACGAAG	CAGATTCCAGACCTGCACAA	60
TBX6	CCTGTGTCTTTCCATCGTGTC	GCCCGAACTAGGTGTATGC	58
NOTO	AAAGCCTCCTCAACCAGAAG	GAGTCTGTGCCTCCAATCAT	60
TBXT	CCTGAGACCCAGTTCATAGC	GTTCCTCCATCATCTCTTTGTG	64
FOXA2	TACGTGTTCATGCCGTTCAT	CGACTGGAGCAGCTACTATGC	64
SOX9	CAAGCTCTGGAGACTTCTGAACG	CCGTTCTTCACCGACTTCCT	60
SOX5	CCTCAAAGCCTCTGTCCCAG	TGCCTTGGTGACAGCATCAT	60
SOX6	AACAACGGCAGCAAATGGAC	TGGATCTGTTGCTGCAGGAG	60
NOGGIN	GGAGGAAGTTACAGATGTGGCTGT	CACTCGGAAATGATGGGGTACTG	63.5
SHH	GCGATTTAAGGAACTCACCC	ACTTGTCCTTACACCTCTGAG	61.5
COL2A1	AGGGCCAGGATGTCCGGCA	GGGTCCCAGGTTCTCCATCT	63
ACAN	CAACTACCCGGCCATCC	GATGGCTCTGTAATGGAACAC	64
mScarlet	CACCAACTTCCCTCCTGACG	GGTGTAGTCCTCGTTGTGGG	60
GAPDH	CAAGATCATCAGCAATGCCT	CAGGGATGATGTTCTGGAGAG	60
YWHAZ	ACTTTTGGTACATTGTGGCTTCAA	CCGCCAGGACAAACCAGTAT	64

Data S1. Differential expression gene list, related to Figure 3 and Figure S3. (Excel file)
